# Smart Materials Employed in the Construction Industry: A Systematic Review of Types, Properties, Applications, and Sustainability Performance

**DOI:** 10.3390/ma19122676

**Published:** 2026-06-22

**Authors:** Hugo Martínez Ángeles, Cesar Augusto Navarro Rubio, José Gabriel Ríos Moreno, Ivan Gonzalez-Garcia, José Luis Reyes Araiza, Mariano Garduño Aparicio, Ernesto Chavero-Navarrete, Mario Trejo Perea

**Affiliations:** Facultad de Ingeniería, Universidad Autónoma de Querétaro, Santiago de Querétaro 76010, Mexico; hmartinez15@alumnos.uaq.mx (H.M.Á.); ivan.glez@uaq.mx (I.G.-G.); lreyes@uaq.mx (J.L.R.A.); mariano.garduno@uaq.mx (M.G.A.); echn_7@hotmail.com (E.C.-N.)

**Keywords:** smart materials, construction materials, intelligent infrastructure, sustainable construction, smart buildings

## Abstract

The construction sector is undergoing a rapid transition toward more resilient, sustainable, and digitally connected systems, creating increasing demand for materials capable of providing functions beyond conventional structural performance. In this context, smart materials have emerged as promising solutions due to their ability to respond to mechanical, thermal, chemical, or electromagnetic stimuli through adaptive behaviors such as self-healing, structural sensing, energy regulation, vibration control, and reversible deformation. Despite growing scientific interest, available knowledge remains fragmented across specific material families and isolated application domains. Therefore, this study presents a PRISMA-based systematic review of smart materials in construction using peer-reviewed journal literature indexed in Scopus during the 2021–2026 period. The review examines the principal smart material families currently applied in construction, including self-healing concretes, self-sensing cementitious systems, Shape Memory Alloys (SMA), piezoelectric materials, phase change materials, adaptive coatings, conductive nanocomposites, and multifunctional geopolymers. Their engineering functions, structural and architectural applications, reported performance characteristics, sustainability contributions, digital integration potential, and implementation barriers are comparatively discussed and qualitatively synthesized based on the reviewed literature. The findings indicate that smart materials can improve durability, structural health monitoring, seismic resilience, thermal efficiency, lifecycle performance, and carbon reduction when properly integrated into buildings and infrastructure. However, large-scale adoption remains constrained by high initial costs, manufacturing scalability, regulatory uncertainty, long-term durability validation, and limited market confidence. The review further shows that the greatest future potential lies in combining material intelligence with IoT platforms, artificial intelligence, BIM environments, and digital twins. Overall, smart materials are positioned as strategic enablers of next-generation low-carbon, adaptive, and intelligent construction systems.

## 1. Introduction

The construction sector is undergoing a profound transformation driven by the need to improve sustainability, resilience, operational efficiency, and lifecycle performance of buildings and infrastructure [1,2]. Conventional construction materials have historically been designed to provide structural capacity and durability; however, current engineering demands increasingly require materials capable of responding actively to environmental, mechanical, thermal, or chemical stimuli [3]. In this context, smart materials have emerged as a promising class of advanced materials capable of providing functions beyond those of traditional passive systems [4].

Smart materials can modify one or more of their properties in response to external stimuli such as stress, temperature, humidity, electric fields, magnetic fields, or damage [5,6,7,8]. These adaptive responses enable functionalities including self-healing, self-sensing, vibration control, thermal energy storage, reversible deformation, and environmental regulation [9,10]. As a result, smart materials are increasingly being investigated for use in structural systems, building envelopes, transportation infrastructure, rehabilitation technologies, and intelligent monitoring networks [11,12].

Recent developments in materials science, nanotechnology, and digital engineering have significantly expanded the range of smart materials available for construction applications [13]. These include self-healing concretes, self-sensing cementitious composites, Shape Memory Alloys (SMA), piezoelectric materials, Phase Change Materials (PCMs), conductive nanocomposites, multifunctional geopolymers, smart coatings, and responsive façade systems [14,15]. Their integration into the built environment offers opportunities to reduce maintenance needs, extend service life, improve structural safety, enhance occupant comfort, and lower operational energy demand [16].

In the case of geopolymers in particular, this functional potential converges with circular-economy goals, as these binders can valorize industrial by-products such as red mud, fly ash, and slag as low-carbon precursors, reducing the embodied carbon of construction materials [17].

Among these systems, self-healing materials can autonomously repair cracks and microdamage, thereby increasing durability and reducing repair interventions [18,19]. Self-sensing concretes and conductive composites enable real-time structural health monitoring through changes in electrical resistance under loading [20,21]. SMA provide recentering capability and energy dissipation in seismic applications, while piezoelectric materials allow sensing and localized energy harvesting [22,23]. In parallel, phase change materials and adaptive thermal systems improve building energy efficiency through passive heat storage and thermal regulation [24].

Despite their strong potential, the widespread implementation of smart materials in construction remains limited by several technical, economic, and regulatory barriers [25,26]. Many systems still involve higher initial costs than conventional alternatives, while long-term durability under realistic service conditions is not always fully validated [27]. Additional challenges include manufacturing scalability, compatibility with existing construction practices, lack of design standards, and uncertainty regarding lifecycle cost-effectiveness [28].

Furthermore, the scientific literature remains fragmented. Existing studies frequently focus on isolated material families such as smart concretes, SMA, thermal systems, or sensing technologies, without providing integrated comparisons across structural performance, sustainability benefits, digital connectivity, implementation maturity, and market readiness [29,30,31]. As a consequence, practitioners and researchers still lack a comprehensive framework for evaluating the relative advantages and limitations of different smart material technologies in construction [32].

In response to these gaps, the present study provides several novel contributions. First, it develops a systematic and comparative synthesis of smart materials currently used in construction based on the PRISMA 2020 (Preferred Reporting Items for Systematic Reviews and Meta-Analyses) methodology [33]. Second, it classifies major smart material families according to their functional mechanisms, engineering applications, and maturity level. Third, it evaluates their structural, environmental, economic, and digital integration potential through a multi-dimensional analytical framework. By combining bibliometric evidence with engineering interpretation, this review advances beyond conventional descriptive surveys and provides a strategic perspective for future intelligent construction systems.

Based on the identified research gaps, this study is structured around the following research questions:

- What are the principal families of smart materials currently applied in construction, and how can they be functionally classified?
- What quantitative engineering benefits do smart materials provide in terms of structural performance, sensing capability, thermal efficiency, and durability?
- How are smart materials being integrated into structural and architectural applications?
- What sustainability and carbon-reduction benefits are associated with smart materials across the construction lifecycle?
- What technical, economic, regulatory, and technological trends will shape the future large-scale adoption of smart materials in construction?

Accordingly, this work employs a PRISMA 2020-based systematic review approach [33] to organize the literature selection process through explicit filtering and evaluation stages. The study examines peer-reviewed journal articles retrieved from Scopus for the 2021–2026 period, enabling an updated assessment of current developments, technological directions, and research activity related to smart construction materials.

This review evaluates material families, engineering properties, structural and architectural applications, sustainability contributions, carbon-reduction potential, IoT and digital integration, barriers to implementation, and future trends. The aim is to support the development of resilient, data-driven, and low-carbon buildings and infrastructure based on multifunctional smart material systems.

This paper proceeds as follows. The methodological basis is laid out in Section 2, Materials and Methods, which describes the PRISMA-based protocol together with the search strategy, the screening procedure, the bibliometric techniques, and the framework adopted for comparative analysis. Building on this, Section 3, Results, brings together the main findings on how materials are classified, the ranges of performance observed, their applications, sustainability implications, implementation barriers, and the trends now emerging. Section 4, Discussion, then offers a critical reading of technological maturity, the pathways available for implementation, and the research gaps that warrant priority attention. The paper closes with Section 5, Conclusions, which distills the central insights and sets out recommendations to guide future work on smart construction materials.

## 2. Materials and Methods

### 2.1. PRISMA Systematic Review Protocol

The methodological design of this work followed the PRISMA 2020 framework (Preferred Reporting Items for Systematic Reviews and Meta-Analyses) [33]. This approach was used to organize the review workflow, including literature retrieval, screening procedures, eligibility verification, and synthesis of the selected evidence.

Study selection proceeded through successive filtering stages in which retrieved records were examined until the final analytical dataset was established. Such a review structure is appropriate for smart materials research in construction because the topic integrates heterogeneous material families, multiple engineering functionalities, distinct implementation levels, and cross-cutting sustainability considerations.

To strengthen methodological openness, the review protocol was retrospectively archived in the Open Science Framework (OSF) [34]. The repository documents the search equation, inclusion and exclusion rules, screening sequence, and analytical organization adopted for the present investigation.

Key methodological components of the protocol are reported throughout this section, including the literature retrieval strategy (Section 2.2), study selection procedure (Section 2.3), quality appraisal methodology (Section 2.4), bibliometric component (Section 2.5), and comparative analytical framework (Section 2.6), thereby facilitating procedural traceability and reproducibility.

The completed PRISMA 2020 checklist was submitted as Supplementary Material. Titles, abstracts, and complete articles were evaluated through a staged screening procedure, and records requiring further clarification were reassessed until agreement was achieved. In addition, methodological robustness among the retained studies was examined through a qualitative appraisal addressing reporting transparency, availability of engineering-related indicators, and cross-study comparability (Section 2.4).

### 2.2. Bibliographic Search Approach

Scientific publications were retrieved from the Scopus database, selected as the primary source of literature due to its strong indexing of journals relevant to engineering, materials research, construction, and sustainability studies. The database enables access to multidisciplinary contributions associated with the development and application of smart materials in the built environment.

To define the search strategy, a keyword-based query was developed by integrating terms linked to intelligent materials, adaptive technologies, sensing mechanisms, structural performance, and sustainability-oriented construction practices, with the objective of obtaining a robust dataset of pertinent studies.

The final search query was structured using Boolean operators as follows: TITLE-ABS-KEY((“smart material*” OR “intelligent material*” OR “self-healing concrete” OR “self-sensing concrete” OR “smart concrete” OR “shape memory alloy*” OR piezoelectric* OR “phase change material*” OR geopolymer* OR nanocomposite* OR “smart brick*” OR conductive composite*) AND (construction OR “civil engineering” OR building OR infrastructure) AND (sustainab* OR “structural health monitoring” OR seismic OR “energy efficiency” OR resilience)) AND PUBYEAR > 2020 AND PUBYEAR < 2027 AND (LIMIT-TO(DOCTYPE,“ar”)) AND (LIMIT-TO(SUBJAREA,“ENGI”) OR LIMIT-TO(SUBJAREA,“MATE”)) AND (LIMIT-TO(LANGUAGE,“English”)).

Applying this strategy yielded an initial set of 1207 records, which offered wide coverage of the pertinent scientific literature and, at the same time, kept the search process consistent, precise, and reproducible.

Although Scopus was used as the sole database, this choice is well supported for built-environment research: approximately 99% of journals indexed in Web of Science are also indexed in Scopus [35], so the two indices largely overlap. In addition, two resources frequently recommended for engineering literature, ScienceDirect and Engineering Village (Compendex), are Elsevier platforms whose journal content is substantially captured within Scopus; using them as separate sources would introduce publisher-related redundancy rather than meaningfully broaden coverage.

Future studies may further broaden coverage by incorporating Dimensions, which provides among the most exhaustive journal coverage of the major indices, and by cross-validating results against Web of Science.

### 2.3. Study Selection Process Based on PRISMA 2020 Methodology

**Figure 1:**
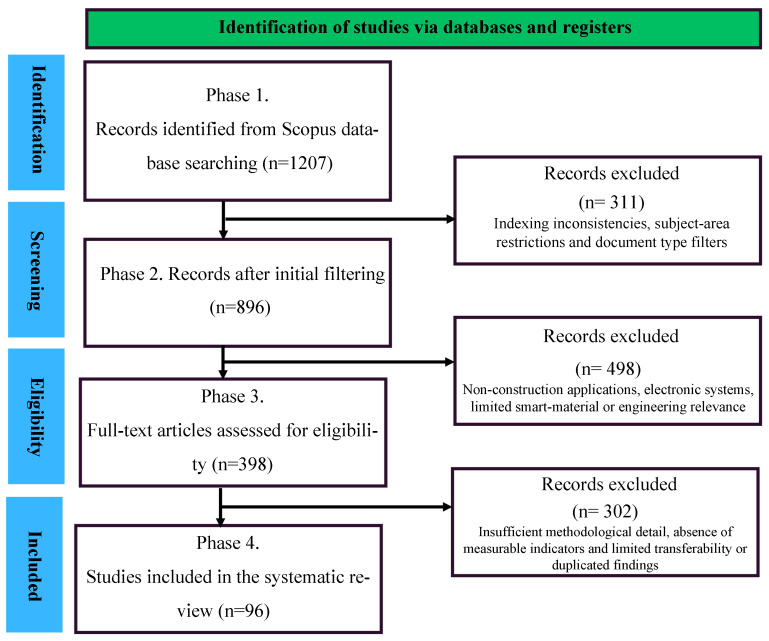
PRISMA flow diagram of the identification, screening, eligibility, and inclusion process of studies retrieved from the Scopus database for the systematic review on smart materials in construction, including the principal exclusion criteria applied at each selection stage.

Guided by the PRISMA methodology, the selection of studies advanced through four successive phases (Figure 1).

In the identification phase, a total of 1207 records were retrieved from the Scopus database using the predefined search strategy. Preliminary filtering was subsequently applied to remove records affected by indexing inconsistencies, publications outside the selected language and subject-area restrictions, non-article document types, and records not satisfying the predefined eligibility filters. As a result, 311 records were excluded and 896 studies advanced to title and abstract screening.

During the screening phase, titles and abstracts were evaluated according to thematic relevance, engineering applicability, and alignment with the objectives of this review. A total of 498 records were excluded because they were unrelated to construction or built-environment applications, focused primarily on biomedical, electronic, or non-civil-engineering systems, lacked a direct connection with smart materials, or provided insufficient engineering relevance for comparative assessment. Consequently, 398 studies were retained for full-text evaluation.

In the eligibility phase, the full texts of the remaining studies were examined to verify methodological transparency, scientific robustness, relevance to construction practice, and availability of sufficiently comparable engineering indicators. At this stage, 302 studies were excluded due to insufficient methodological detail, absence of measurable or interpretable performance indicators, duplicated findings across closely related publications, highly specific case-study scope limiting broader applicability, or limited transferability to construction practice.

Finally, in the inclusion phase, 96 studies satisfied all predefined selection criteria and were incorporated into the final systematic review. These studies provided the principal evidence base for the comparative assessment of smart materials applied in construction, infrastructure, sensing systems, sustainability, adaptive performance, and resilient engineering applications.

The narrowing of records from the original search output to the final study set emerged from successive filtering decisions applied during identification, screening, and eligibility assessment stages. This progression reflects a controlled evidence-selection strategy intended to maintain coherence in scope, comparability of information, and methodological consistency among the retained publications.

Within the PRISMA workflow, the final set of 96 studies represents the core analytical dataset used for comparative synthesis. References appearing outside this corpus were employed selectively to strengthen conceptual grounding, support methodological explanations, justify database-related decisions, and contextualize the interpretation of results.

### 2.4. Study Quality and Robustness Assessment

The retained publications were examined through a structured evaluation process intended to characterize evidence reliability and methodological soundness. Five assessment dimensions were considered:

- Explicitness and clarity of the methodological description;
- Presence of quantitative engineering-related metrics;
- Degree of consistency and cross-study comparability of reported findings;
- Alignment with smart-material applications in the construction domain;
- Relevance to sustainability, resilience, sensing functions, or performance enhancement.

For each dimension, studies were classified using a three-tier rating scheme (adequate, partial, or insufficient). The combined outcome of these ratings was subsequently used to assign an overall evidence reliability category (high, moderate, or low) for each included study.

Formal quantitative risk-of-bias instruments (e.g., RoB 2, ROBINS-I) were not applied because they are designed for clinical intervention studies with defined comparators and outcomes, and are not transferable to the experimental materials-engineering studies synthesized here. The structured appraisal described above was adopted instead, providing a transparent and reproducible basis for assessing evidence quality across heterogeneous study types.

Studies rated as insufficient in methodological transparency or in the availability of comparable data were excluded during the eligibility phase, while the reliability level of the retained studies was considered when weighting their evidence in the comparative assessment.

### 2.5. Bibliometric Analysis

**Figure 2:**
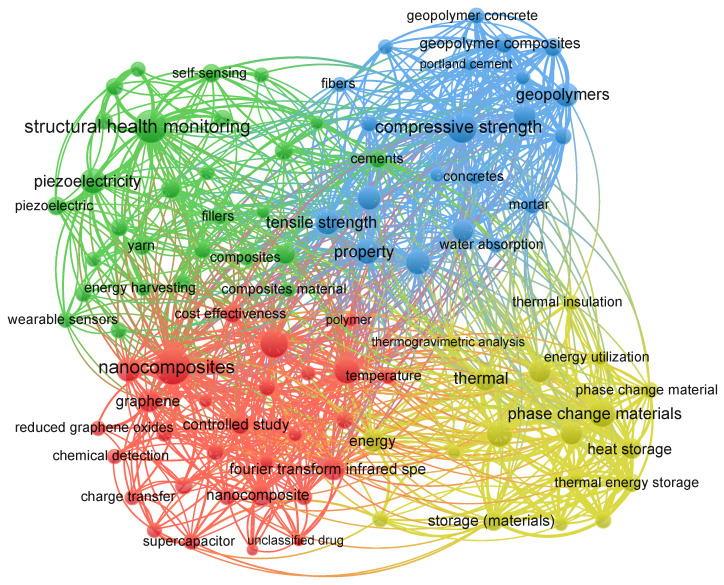
Thematic structure of smart materials research in construction, visualized as a keyword co-occurrence network. In this map, keyword frequency is encoded by node size, co-occurrence strength by link thickness, and thematic clusters by color, all derived through VOSviewer. Strong connections emerge among nanotechnology, structural health monitoring, geopolymer systems, thermal energy storage, and multifunctional construction materials. Source: authors’ own elaboration, drawing on bibliometric data extracted from Scopus (2021–2026).

Figure 2 shows the bibliometric keyword co-occurrence network related to smart materials in construction, generated using VOSviewer software (version 1.6.20). The map illustrates relationships among terms based on occurrence frequency, thematic proximity, and co-occurrence strength, enabling the identification of major research areas and interdisciplinary connections within the field.

The network reveals a broad and highly interconnected research landscape, confirming that smart materials in construction have evolved into a multidisciplinary domain integrating structural engineering, materials science, nanotechnology, thermal management, and digital monitoring systems.

Several thematic clusters can be identified. A first cluster, centered on nanocomposites, graphene, reduced graphene oxides, and related characterization techniques, reflects the growing relevance of nanoscale material engineering. This cluster is strongly associated with conductivity enhancement, crack sensing, multifunctionality, and advanced composite performance. The prominence of graphene-based terms confirms the increasing use of carbon nanomaterials to improve durability, self-sensing capacity, and electromechanical response.

A second cluster is organized around structural health monitoring, self-sensing, piezoelectricity, piezoelectric, and energy harvesting. This thematic group highlights the transition from passive construction systems toward intelligent infrastructure capable of real-time monitoring, damage detection, vibration response measurement, and decentralized energy generation. The presence of sensing-related nodes confirms that embedded diagnostics has become one of the most dynamic research directions in the field.

A third major cluster is associated with cementitious and geopolymeric materials, where terms such as geopolymers, geopolymer concrete, geopolymer composites, compressive strength, cements, mortar, and water absorption appear as dominant nodes. This indicates that alternative binders and low-carbon cementitious systems are increasingly integrated into smart material research while maintaining strong emphasis on conventional engineering performance indicators such as strength and durability.

A fourth thematic cluster is strongly linked to thermal and energy applications, dominated by phase change materials, phase change material, thermal energy storage, heat storage, and thermal insulation. This confirms the rapid expansion of adaptive envelopes, intelligent walls, PCM-enhanced bricks, and passive energy-saving systems designed to reduce operational energy demand and improve indoor thermal comfort.

The coexistence of these clusters demonstrates that current research no longer evaluates construction materials solely according to mechanical strength. Instead, smart materials are increasingly expected to combine structural reliability with additional functionalities such as sensing, self-repair, thermal regulation, energy harvesting, and sustainability improvement.

The relative centrality of terms such as nanocomposites, structural health monitoring, compressive strength, geopolymers, and phase change materials suggests that future innovation in the sector will likely emerge from hybrid solutions that combine intelligent functionality with sustainability and structural performance.

From a thematic perspective, the network also indicates an ongoing transition from conventional cement-based material optimization toward multifunctional systems emphasizing decarbonization, adaptive behavior, resilience, and digital integration. This trend is consistent with the increasing role of smart materials in next-generation buildings and infrastructure.

The included publications exhibited marked variability in experimental scale, environmental conditions, constituent materials, and testing protocols, limiting the feasibility of performing a conventional meta-analysis. For this reason, evidence integration relied on a qualitative synthesis framework combined with cross-study comparison to support consistent interpretation despite methodological diversity.

Within this methodological structure, bibliometric analysis serves a supporting rather than central analytical role. It is used to outline patterns of research activity, thematic concentration, and technological evolution in the field, while the principal conclusions of the review derive from the comparative examination of the selected studies.

### 2.6. Analytical and Conceptual Framework for Comparative Assessment

To compare smart materials used in construction on a consistent basis, this review builds a conceptual and analytical framework that links three aspects often examined in isolation. The first concerns how a material performs structurally and functionally; the second addresses its environmental efficiency throughout its service life; and the third reflects how mature the technology is and how readily it can be deployed in practice. Bringing these aspects together provides methodological consistency across material classification, engineering metrics, and sustainability criteria.

This framework is not intended as a unified empirical aggregation model, but rather as an integrative analytical structure designed to harmonize interpretation across heterogeneous studies. Given the substantial variability in material composition, functional mechanisms, specimen geometry, testing procedures, environmental exposure, and reported data across the reviewed literature, direct quantitative aggregation was not considered methodologically robust. Consequently, the analytical indicators adopted in this section [19,36,37,38,39,40,41,42,43,44,45] are employed exclusively as comparative interpretation tools rather than predictive models.

The principal indicators used to support systematic comparison across heterogeneous smart material systems are defined as follows:

(1) $$f_{c}=\frac{P_{max}}{A}$$

where $f_{c}$ represents compressive strength (MPa), $P_{max}$ is the maximum applied load, and *A* is the loaded area.

(2) $$HE=\frac{R_{h}}{R_{0}}\times 100$$

where HE denotes healing efficiency (%), $R_{h}$ corresponds to recovered performance after healing, and $R_{0}$ represents original performance.

(3) $$FCR=\frac{\Delta R}{R_{0}}$$

where FCR is the fractional change in electrical resistance during loading.

(4) Q=mL

where *Q* corresponds to latent heat storage, *m* is PCM mass, and *L* is latent heat.

(5) $$CF=\frac{CO_{2}}{f_{c}}$$

where CF represents carbon intensity normalized by structural strength.

(6) $$LCCE=C_{i}+C_{m}-S_{l}$$

where $C_{i}$ is initial cost, $C_{m}$ represents maintenance cost, and $S_{l}$ denotes lifecycle savings.

(7) TRL=1→9

where increasing values indicate progression from laboratory validation toward market implementation.

Because smart materials differ substantially in composition, scale, service conditions, environmental exposure, and testing methodologies, these formulations should not be interpreted as universal predictive models. Rather, they provide a structured analytical basis for harmonizing heterogeneous evidence and for interpreting performance trends, technological maturity, sustainability considerations, and engineering functionality across diverse smart material systems.

## 3. Results

### 3.1. Classification and Functional Roles of Smart Materials in Construction

Smart materials are increasingly recognized as innovative components in modern construction due to their ability to respond to external stimuli and provide functions beyond those of conventional materials [46,47,48]. Depending on their composition and mechanism, these systems may offer self-healing, structural sensing, adaptive damping, thermal regulation, or reversible deformation [31,49,50]. Their incorporation into buildings and infrastructure is associated with improved durability, resilience, monitoring capability, and energy efficiency [51,52,53]. Therefore, a clear functional classification is essential for understanding their engineering relevance and practical applications.

Table 1 presents a taxonomic classification of the principal smart material families currently reported in construction research, including operating principles, representative examples, main applications, and supporting references.

Table 1 shows that smart materials can be grouped according to the engineering function they provide. Self-healing systems and smart concretes are mainly focused on durability enhancement and condition monitoring [30,48,49]. Piezoelectric and conductive materials are strongly linked to structural health monitoring, enabling real-time diagnostics and localized energy harvesting [46,55,62].

Shape memory and rheological materials are particularly relevant for seismic engineering, where adaptive damping, recentering behavior, and vibration mitigation are essential [31,56,60]. In contrast, thermochromic and adaptive thermal materials are associated with building envelope optimization, reducing thermal loads and improving occupant comfort [50,57,58]. Multifunctional geopolymers additionally contribute to sustainability by combining low-carbon binders with intelligent functionalities [64,65].

**Figure 3:**
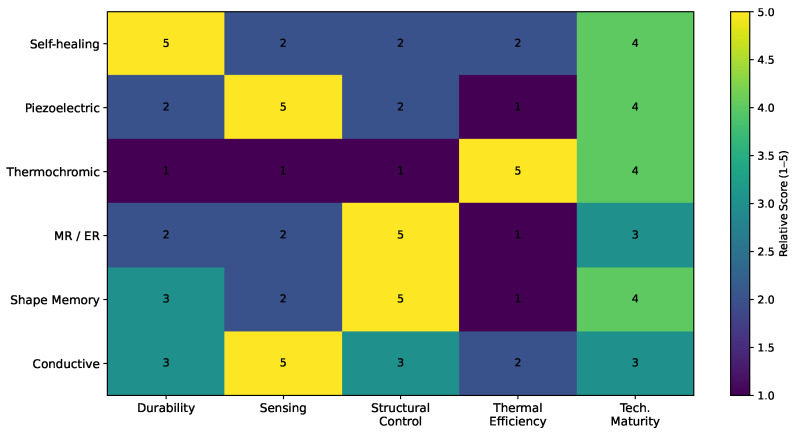
Qualitative comparative assessment of major smart material families used in construction according to five engineering criteria: durability enhancement, sensing capability, structural control potential, thermal efficiency, and technological maturity. Relative intensity levels were assigned from performance trends consistently reported in the reviewed literature to illustrate comparative strengths and application-oriented trade-offs among different smart material systems.

To complement the classification presented in Table 1, Figure 3 provides a qualitative comparison of the principal material families according to five relevant engineering dimensions: durability enhancement, sensing capability, structural control potential, thermal efficiency, and technological maturity.

Figure 3 highlights the multifunctional diversity of smart construction materials. Self-healing systems show the strongest durability benefits [49,54], piezoelectric and conductive materials dominate sensing functions [46,47,62], shape memory and rheological systems are more relevant for structural control [31,46], whereas thermochromic and PCM-based materials provide superior thermal adaptation [50,57]. These findings indicate that future intelligent buildings and infrastructure will likely depend on hybrid combinations of several smart material technologies rather than on a single material solution [47,48].

### 3.2. Main Families of Smart Materials Used in Construction

Smart materials used in the construction industry can be grouped into several technological families according to their dominant response mechanism and engineering function. These materials are designed to react to environmental, mechanical, thermal, or electromagnetic stimuli, enabling advanced capabilities such as self-repair, structural monitoring, seismic control, thermal storage, or adaptive surface behavior [46,48,66]. Their growing implementation reflects the transition from passive infrastructure toward multifunctional and intelligent built environments [47,67,68].

Table 2 summarizes the principal smart material families from a construction implementation perspective, highlighting representative examples, main stimuli, functional responses, and supporting references.

Table 2 shows that smart materials in construction address different engineering challenges depending on the type of stimulus and desired response. Smart concretes are primarily associated with durability enhancement and structural health monitoring, whereas SMA and rheological fluids are more relevant in seismic protection and vibration mitigation [31,46,55,66]. These systems are particularly valuable in resilient infrastructure subjected to dynamic loading [56,61].

Piezoelectric materials stand out for their dual capability of sensing and energy harvesting, making them attractive for self-powered monitoring systems [67,68,71]. In contrast, phase change materials and smart coatings are mainly linked to building energy efficiency through passive thermal control and adaptive surface behavior [48,51,69]. Smart geopolymers combine multifunctionality with sustainability by incorporating low-carbon binders and intelligent responses [49,64].

**Figure 4:**
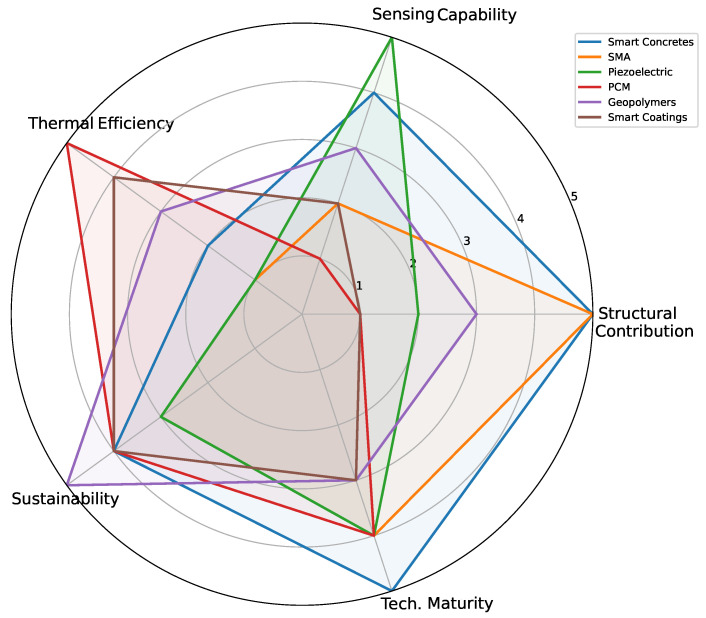
Qualitative multifunctional comparison of major smart material families used in construction according to structural contribution, sensing capability, thermal efficiency, sustainability potential, and technological maturity. Relative scores were assigned from trends consistently reported in the reviewed literature to illustrate comparative strengths and application-oriented trade-offs.

To complement the classification presented in Table 2, Figure 4 provides a qualitative multifunctional comparison of the principal material families according to five relevant engineering criteria: structural contribution, sensing capability, thermal efficiency, sustainability potential, and technological maturity.

Figure 4 highlights that no single material family maximizes all engineering criteria simultaneously. Smart concretes and SMA exhibit strong structural relevance [61,66], piezoelectric materials dominate sensing functions [46,71], PCMs and smart coatings perform better in thermal efficiency [48,69], while geopolymers stand out in sustainability [49,64]. These findings suggest that future intelligent buildings and infrastructure will likely integrate multiple smart material technologies in hybrid systems [47,67].

### 3.3. Functional Properties and Quantitative Performance Ranges

The practical relevance of smart materials in construction depends not only on their responsive behavior, but also on the measurable improvements they provide under real engineering conditions. Recent studies report significant gains in sensing precision, fracture resistance, corrosion durability, thermal performance, and environmental sustainability [48,64,67,71]. These quantitative indicators are essential for evaluating the feasibility of smart materials as alternatives to conventional systems [49,68,72].

Table 3 summarizes the principal functional properties and reported performance ranges of smart materials currently applied in construction environments.

Table 3 indicates that smart materials can provide substantial and measurable engineering benefits. In sensing applications, the detection of microstrains as low as 10 µϵ demonstrates the potential of self-sensing concretes and ultra-high-performance composites for structural health monitoring [48,68,71]. Similarly, conductivity values near 10 S/m confirm the feasibility of conductive masonry and cementitious systems for embedded diagnostics [64,69].

Mechanical and durability improvements are also significant. Fracture toughness gains of approximately 50% suggest that fiber-reinforced and nano-modified materials can substantially improve crack resistance and post-cracking behavior [48,73]. In addition, corrosion resistance improvements near 40% highlight the durability advantages of SMA and intelligent protective coatings in aggressive environments [31,48].

Thermal and environmental indicators further support the value of smart materials in sustainable construction. PCM-integrated walls can reduce heat flow by approximately 45%, while advanced manufacturing methods combined with green materials may lower embodied CO_2_ emissions by 40–60% [67,72,74]. These findings demonstrate that smart materials simultaneously address structural performance, operational efficiency, and sustainability objectives [49,64].

**Figure 5:**
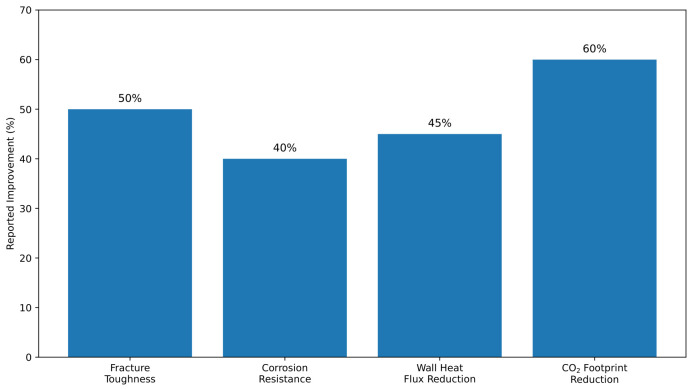
Comparative representation of reported quantitative performance gains of smart materials used in construction, including fracture toughness improvement, corrosion resistance, wall heat flux reduction, and carbon footprint mitigation. Values were normalized from ranges reported in the reviewed literature for visual comparison.

To complement the quantitative results presented in Table 3, Figure 5 illustrates the relative magnitude of the principal reported performance improvements across different engineering criteria.

Figure 5 shows that the highest reported relative benefits are associated with environmental impact reduction and mechanical enhancement, while corrosion mitigation and thermal control also exhibit strong practical relevance [67,72,73,74]. Overall, these results confirm that smart materials can generate measurable improvements across multiple dimensions of construction performance [48,49].

### 3.4. Structural and Architectural Applications of Smart Materials

The value of smart materials in construction is best demonstrated through their direct application in structural and architectural systems. These materials are increasingly used to improve safety, durability, energy efficiency, and lifecycle performance of buildings and infrastructure [48,66,67]. Depending on the engineering objective, smart materials can operate as sensing elements, adaptive control devices, self-repair systems, or passive environmental regulators [31,46,49].

Table 4 summarizes the principal structural and architectural applications of smart materials currently reported in the literature.

Table 4 shows that smart materials are already addressing both structural and architectural challenges. In bridges and buildings, self-sensing concretes, optical fibers, and piezoelectric sensors enable continuous structural health monitoring, improving preventive maintenance and operational safety [46,68,71]. Likewise, SMA and rheological devices are highly valuable in seismic protection systems due to their adaptive damping and recentering capacity [31,56,66].

Architectural applications are mainly associated with energy efficiency and occupant comfort. PCMs, smart façades, and intelligent masonry systems can regulate thermal loads and daylight conditions, reducing HVAC demand [48,67,69]. In addition, self-healing concrete and smart strengthening systems contribute to lifecycle extension of aging infrastructure while minimizing invasive interventions [49,61,70].

**Figure 6:**
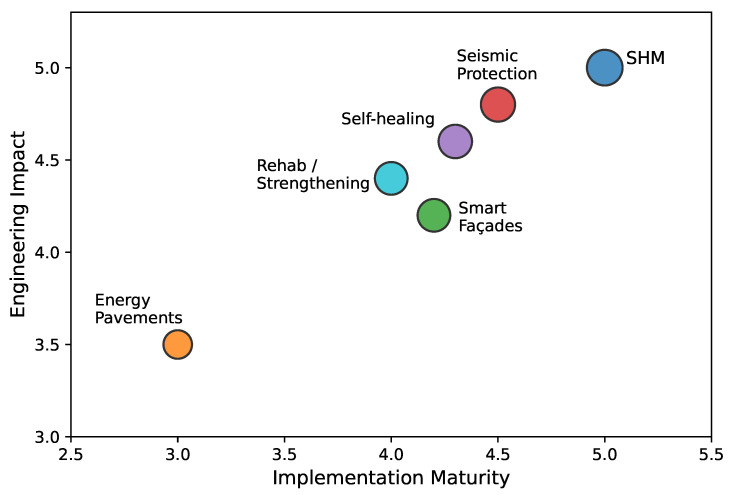
Qualitative comparison of major smart material application domains in construction according to engineering impact and implementation maturity. Bubble size represents the relative multifunctional potential of each application area based on trends reported in the reviewed literature.

To complement the applications summarized in Table 4, Figure 6 illustrates the relative positioning of major application domains according to engineering impact and implementation maturity.

Figure 6 indicates that structural health monitoring and climate-responsive envelopes currently show the highest implementation maturity, while seismic protection and autonomous healing systems exhibit strong engineering impact [48,49,66,71]. Energy-harvesting pavements remain an emerging application with promising multifunctional benefits but comparatively lower deployment maturity [56,67]. These results suggest that smart materials are progressively transitioning from experimental concepts to practical construction technologies [46,68].

### 3.5. Smart Concretes and Self-Sensing UHPC Systems

Concrete remains the most widely used construction material worldwide; therefore, incorporating intelligent functionalities into cementitious systems has become a major research priority [48,49,66]. Smart concretes are designed to provide autonomous responses such as crack healing, structural sensing, or environmental adaptation while preserving structural capacity [61,68,71]. Recent developments in ultra-high-performance concrete (UHPC) and geopolymer technologies have further expanded the multifunctional potential of intelligent cement-based materials [64,68,73].

Table 5 summarizes the principal categories of smart concretes currently reported in the literature, including their dominant mechanisms, advantages, limitations, and representative references.

Table 5 shows that intelligent concrete technologies can be grouped according to their dominant function: durability enhancement, sensing capability, ultra-high mechanical performance, or sustainability. Self-healing concretes are primarily focused on autonomous crack sealing and maintenance reduction, while self-sensing concretes enable distributed monitoring without relying on external sensors [49,66,71,73].

Self-sensing UHPC combines very high compressive strength with sensitivity to damage evolution, making it particularly attractive for bridges, tunnels, and critical infrastructure [68]. Meanwhile, smart geopolymer concretes provide a promising low-carbon alternative by combining waste valorization with conductive or adaptive functionalities [49,64]. Despite these advantages, cost, industrial scalability, and standardization remain key barriers to widespread implementation [48,66].

**Figure 7:**
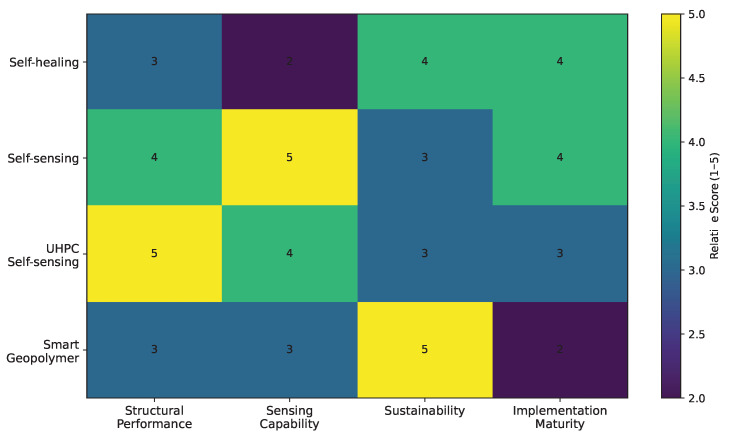
Qualitative comparison of major smart concrete systems according to structural performance, sensing capability, sustainability contribution, and implementation maturity. Relative scores were derived from trends reported in the reviewed literature to illustrate the comparative positioning of advanced cementitious technologies.

To complement the classification presented in Table 5, Figure 7 compares the principal smart concrete systems according to four strategic criteria: structural performance, sensing capability, sustainability contribution, and implementation maturity.

Figure 7 indicates that self-sensing UHPC provides the strongest structural performance [68], conventional self-sensing concrete leads in sensing functionality [71,73], smart geopolymers dominate sustainability indicators [49,64], and self-healing concrete exhibits strong lifecycle advantages through durability enhancement [66,70]. These findings suggest that future intelligent infrastructure will likely combine several cementitious smart technologies depending on project priorities [48,61].

### 3.6. Sustainability and Environmental Performance

Sustainability has become a central driver in the adoption of smart materials within the construction industry. Beyond structural benefits, many intelligent materials contribute to reducing embodied carbon, minimizing waste generation, lowering operational energy demand, and improving long-term resource efficiency [48,49,64,72]. These strategies are particularly relevant as the built environment remains one of the largest contributors to global emissions and material consumption [67,69,74].

Table 6 summarizes the principal sustainability strategies associated with smart materials and their reported environmental or economic benefits.

Table 6 shows that smart materials contribute to sustainability through both material substitution and operational optimization. Replacing ordinary Portland cement (OPC) with geopolymers or supplementary cementitious materials can significantly reduce embodied emissions [49,64,72]. Likewise, additive manufacturing technologies improve material efficiency by reducing waste and enabling optimized geometries [48,72].

Operational performance is also enhanced through PCM-integrated systems and adaptive building envelopes, which can substantially reduce heating and cooling demand [48,67,69]. At the urban scale, durable smart materials and noise-control technologies support greener and more livable cities by lowering maintenance frequency and improving environmental comfort [31,69].

Artificial intelligence further amplifies these benefits by optimizing design, material allocation, and lifecycle decision-making [74]. The integration of AI with smart materials suggests a new pathway toward data-driven sustainable construction systems with improved cost-effectiveness and reduced environmental burden [48,74].

**Figure 8:**
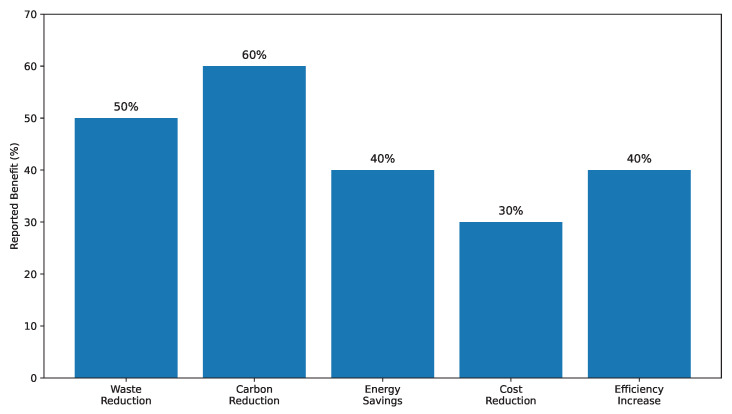
Comparative representation of reported sustainability benefits of smart construction technologies, including waste reduction, carbon footprint mitigation, operational energy savings, cost reduction, and efficiency improvement. Values were derived from ranges reported in the reviewed literature for comparative visualization.

To complement the results summarized in Table 6, Figure 8 compares the reported magnitude of key environmental and economic benefits associated with smart construction technologies.

Figure 8 indicates that the largest reported gains are associated with carbon footprint reduction and waste minimization, followed by substantial operational energy savings and economic optimization through AI-assisted systems [48,72,74]. These findings confirm that smart materials can play a strategic role in decarbonizing and modernizing the construction sector [49,64,67].

### 3.7. Smart Materials for Carbon Reduction and Low-Carbon Performance

Reducing carbon emissions across the construction lifecycle has become one of the main drivers for innovation in smart materials. Beyond improving structural or functional performance, several emerging material families directly contribute to decarbonization through lower embodied energy, reduced cement consumption, operational energy savings, circular resource use, or enhanced durability [48,49,75,76]. In parallel, specific technical properties such as biogenic carbon storage, recyclability, and self-healing behavior further strengthen the environmental value of these materials [77,78,79].

Table 7 summarizes the principal smart material families associated with carbon mitigation in construction, including representative examples, dominant reduction mechanisms, and the lifecycle stages where their benefits are most relevant.

Table 8 complements the material-family perspective by summarizing the principal technical properties that enable carbon reduction and circular performance in smart construction systems.

Table 7 and Table 8 show that decarbonization in construction can be achieved through multiple complementary pathways. Advanced concretes, geopolymers, and recycled materials mainly reduce embodied emissions during production, whereas PCMs and smart envelopes target operational carbon through lower heating and cooling demand [48,76,93,96].

Bio-based materials stand out because they combine low embodied energy with temporary carbon sequestration during service life [75,77,85]. In parallel, self-healing systems contribute indirectly by extending durability and reducing replacement frequency, while recyclable and prefabricated systems improve circularity and reduce construction waste [49,78,79,98].

These findings indicate that no single solution dominates all sustainability dimensions. Effective low-carbon construction will likely depend on combining material substitution, performance enhancement, operational efficiency, and circular economy strategies [72,82,92].

**Figure 9:**
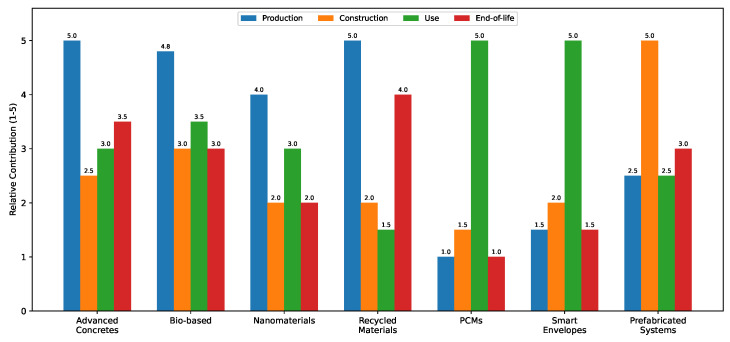
Qualitative comparison of major smart material families contributing to carbon reduction in construction according to lifecycle stages: production, construction, operational use, and end-of-life. Relative contribution levels were assigned from trends consistently reported in the reviewed literature.

To complement the synthesis presented in Table 7 and Table 8, Figure 9 compares the relative contribution of the main smart material families across production, construction, operational use, and end-of-life stages.

Figure 9 indicates that advanced concretes and recycled materials dominate production-stage mitigation, PCMs and smart envelopes lead operational savings, and prefabricated smart systems provide the strongest construction-stage efficiency [76,93,95,98]. Overall, lifecycle-oriented combinations of multiple smart material strategies are required for meaningful decarbonization of the built environment [48,72,75].

### 3.8. Digital Integration, Sensors, and IoT

The performance of smart materials can be significantly enhanced when combined with digital technologies such as sensing networks, artificial intelligence, and digital twins. While smart materials provide responsive physical behavior, digital platforms enable real-time monitoring, predictive analysis, and lifecycle optimization [48,66,74]. This convergence is accelerating the transformation of conventional buildings into intelligent and connected infrastructure systems [49,67,99].

Table 9 summarizes the principal enabling digital technologies currently associated with smart materials in construction.

Table 9 shows that digital technologies act as amplifiers of smart material performance. IoT-enabled structural health monitoring systems allow embedded sensors to continuously transmit data regarding strain, vibration, humidity, or damage evolution. This improves maintenance planning and reduces the risk of unexpected failures [46,48,67].

Artificial intelligence and machine learning contribute by identifying optimal materials, predicting deterioration, and improving operational efficiency [49,74]. In parallel, digital twin and BIM platforms enable virtual representations of smart structures, supporting design optimization, performance simulation, and lifecycle management [66,74,99].

The integration of smart materials with digital ecosystems suggests a shift from isolated responsive components toward autonomous and data-driven infrastructure. This evolution is expected to play a major role in future smart cities and resilient construction systems [48,67].

**Figure 10:**
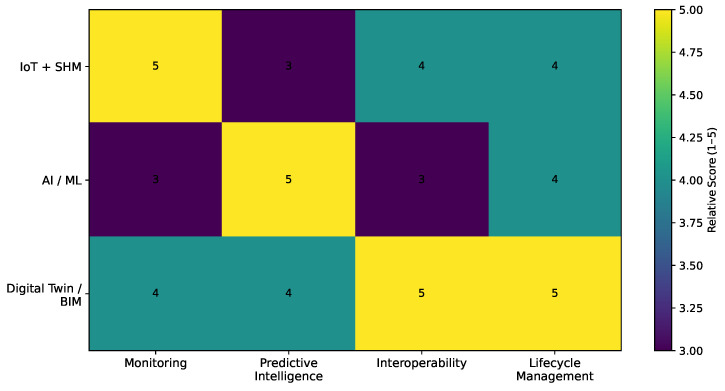
Qualitative comparison of digital support technologies integrated with smart materials in construction according to monitoring capability, predictive intelligence, interoperability, and lifecycle management potential. Relative scores were assigned from trends reported in the reviewed literature for comparative visualization.

To complement the results summarized in Table 9, Figure 10 illustrates the relative contribution of major digital technologies to monitoring capability, predictive intelligence, and lifecycle management in smart construction environments.

Figure 10 indicates that IoT platforms dominate real-time monitoring functions, AI/ML systems provide the strongest predictive intelligence, and digital twin/BIM environments lead lifecycle management and interoperability performance [48,74,99]. These findings confirm that the full potential of smart materials is achieved when physical intelligence is combined with digital intelligence [49,66].

### 3.9. Technical and Economic Challenges

Despite their promising multifunctional benefits, the widespread implementation of smart materials in construction is still constrained by several technical and economic barriers. Many intelligent systems remain more expensive than conventional alternatives, while issues related to scalability, compatibility, and long-term durability continue to limit market penetration [31,64,66,73]. Addressing these challenges is essential for transitioning smart materials from niche applications to mainstream construction practice [48,49,68].

Table 10 summarizes the principal technical and economic challenges currently affecting the adoption of smart materials in the construction sector.

Table 10 shows that economic barriers remain among the most influential constraints for smart material adoption. High initial costs are particularly relevant for SMA, conductive nanomaterials, and advanced sensing systems, reducing their feasibility in cost-sensitive projects [66,68,73]. This issue is especially critical in developing markets where lifecycle benefits are often secondary to upfront investment decisions [31,74].

Manufacturing scalability is another important limitation. Advanced materials such as self-sensing UHPC and multifunctional geopolymers frequently depend on controlled production processes, specialized additives, or non-standardized formulations, making industrial deployment more complex [49,64,68]. In addition, compatibility with traditional construction methods remains a challenge, especially for retrofitting or in situ applications [48,67].

Long-term durability uncertainty also affects confidence in these technologies. Potential degradation of nanomaterials, corrosion susceptibility of metallic smart alloys, and aging of responsive polymers require further validation under real service conditions [31,64]. Therefore, broader adoption will depend not only on performance improvements, but also on cost reduction, standardized production, and proven lifecycle reliability [48,66].

**Figure 11:**
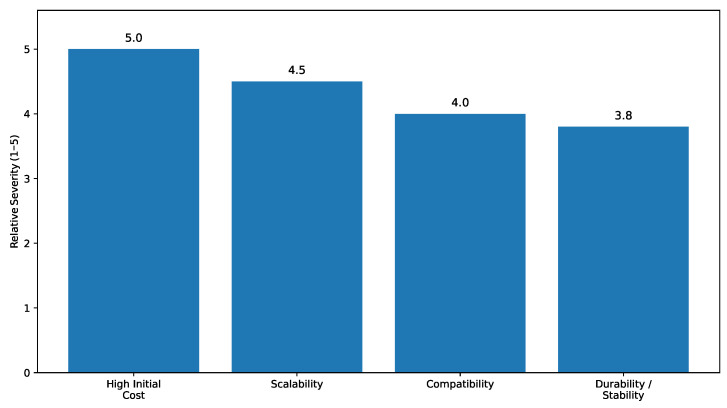
Qualitative comparison of the principal barriers affecting adoption of smart materials in construction, including initial cost, scalability, compatibility, and long-term durability uncertainty. Relative severity levels were assigned from trends consistently reported in the reviewed literature.

To complement the results summarized in Table 10, Figure 11 compares the relative severity of the main barriers currently limiting smart material deployment in construction.

Figure 11 indicates that high initial cost and industrial scalability are currently the most restrictive factors, followed by compatibility challenges and durability uncertainty [49,66,68]. These findings suggest that future research should focus not only on material innovation, but also on manufacturability, standardization, and economic feasibility [48,74].

### 3.10. Regulatory Challenges and Market Acceptance

Beyond technical performance, the successful adoption of smart materials in construction also depends on regulatory readiness, operational security, and stakeholder confidence. Many intelligent construction technologies are advancing faster than current building codes and certification frameworks, creating uncertainty for designers, contractors, and regulators [31,49,64,73]. In addition, the digitalization associated with embedded sensing systems introduces new cybersecurity concerns [48,74].

Table 11 summarizes the principal regulatory and market acceptance barriers currently affecting smart material deployment in the construction sector.

Table 11 shows that regulatory limitations remain one of the strongest non-technical barriers to smart material implementation. The absence of standardized codes for SMA, geopolymer systems, and structural health monitoring technologies complicates approval processes, performance verification, and insurance acceptance [31,49,64]. As a result, innovative materials often face delays in certification and commercial deployment [72,73].

Digitalization introduces an additional layer of risk. Smart materials integrated with sensors, wireless communication, and automated monitoring systems may become vulnerable to cyberattacks or data manipulation, potentially compromising operational reliability [48,74]. This is particularly relevant for critical infrastructure where false readings or system interruption could affect safety decisions [48].

Market acceptance is also influenced by uncertainty regarding long-term durability, maintenance costs, and return on investment. Developers and end users may perceive smart materials as experimental or financially risky, even when lifecycle benefits are favorable [31,73]. Therefore, wider adoption will require not only better regulations, but also clearer business cases and proven field performance [72,74].

**Figure 12:**
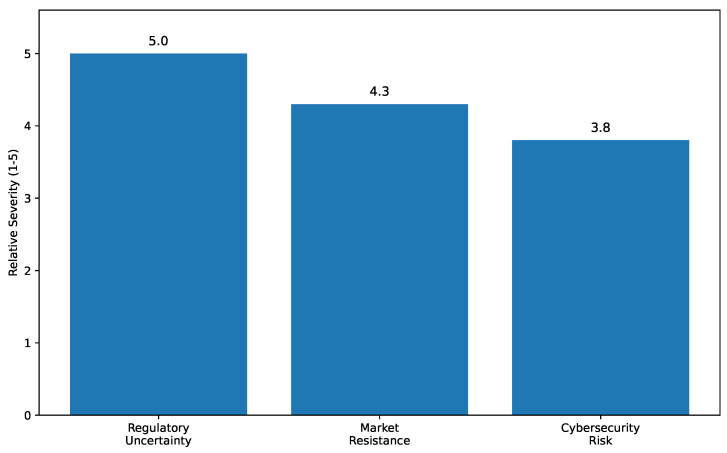
Qualitative comparison of major regulatory and market acceptance barriers affecting smart materials in construction, including lack of standards, cybersecurity risks, and stakeholder resistance. Relative severity levels were assigned from trends consistently reported in the reviewed literature.

To complement the results summarized in Table 11, Figure 12 compares the relative severity of the main regulatory and acceptance challenges limiting smart material deployment in construction.

Figure 12 indicates that regulatory uncertainty remains the most restrictive barrier, followed by market resistance and cybersecurity concerns [31,48,73]. These findings suggest that future progress will depend not only on technological innovation, but also on standards development, secure digital infrastructure, and increased confidence among industry stakeholders [72,74].

### 3.11. Impacts on Structural Sustainability: Seismic Resilience and Lifecycle Performance

Structural sustainability extends beyond environmental indicators and includes resilience, lifecycle efficiency, maintainability, and social safety. In this context, smart materials can substantially improve the long-term performance of buildings and infrastructure by reducing damage during extreme events, minimizing maintenance interventions, and extending service life [31,49,61,66]. These benefits are especially relevant for seismic regions and aging asset portfolios [56,68,70].

Table 12 summarizes the principal contributions of smart materials to structural sustainability from the perspectives of seismic resilience and lifecycle performance.

Table 12 shows that smart materials contribute to sustainability through both preventive and restorative strategies. In seismic applications, SMA and adaptive damping devices can reduce structural damage and post-event downtime, improving safety and community resilience after earthquakes [31,56].

Lifecycle benefits are also significant. Self-healing concretes and structural health monitoring systems reduce the need for frequent inspections, emergency repairs, and unexpected shutdowns [49,66,68]. These technologies therefore improve asset reliability while lowering total ownership costs over time [61,70].

Smart materials are also valuable in rehabilitation projects, especially for historic or sensitive structures where intrusive interventions should be minimized. SMA-based retrofitting systems and intelligent composites can enhance capacity while preserving architectural value and reducing material consumption [31,56].

**Figure 13:**
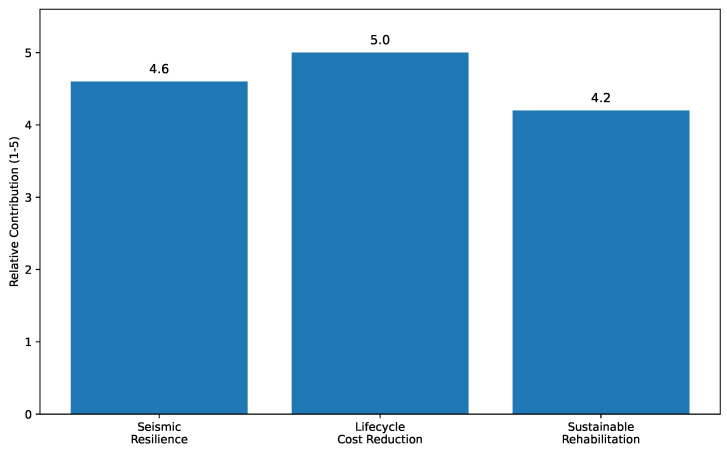
Qualitative comparison of major smart material contributions to structural sustainability in construction, considering seismic resilience, lifecycle cost reduction, and sustainable rehabilitation potential. Relative contribution levels were assigned from trends consistently reported in the reviewed literature.

To complement the results summarized in Table 12, Figure 13 compares the relative contribution of smart material solutions to seismic resilience, lifecycle savings, and sustainable rehabilitation.

Figure 13 indicates that lifecycle cost reduction represents the strongest recurring benefit, followed by seismic resilience and sustainable rehabilitation [31,49,66]. These findings confirm that smart materials support structural sustainability not only through environmental gains, but also through safer, longer-lasting, and more resource-efficient infrastructure systems [56,61,68].

### 3.12. Emerging Trends in Smart Materials for Construction

The next generation of smart construction materials is evolving beyond passive multifunctionality toward adaptive, programmable, and digitally integrated systems. Recent advances in additive manufacturing, embedded electronics, responsive surfaces, and time-dependent materials are opening new possibilities for intelligent buildings and infrastructure [48,51,67,99]. These trends indicate that future construction systems may actively interact with users and environmental conditions [48,69].

Table 13 summarizes the principal emerging trends currently identified in the literature and their expected impact on the construction sector.

Table 13 shows that future smart materials are increasingly associated with programmable behavior and systems-level intelligence. Smart bricks produced through additive manufacturing may combine thermal regulation, sensing capability, and modular assembly, enabling faster and more efficient construction methods [48,51].

Acoustic metasurfaces represent another promising direction, especially in dense urban environments where noise pollution is a growing challenge. These engineered surfaces can dynamically manipulate sound propagation, improving indoor and outdoor acoustic comfort [69].

The concept of 4D structures goes further by incorporating time-dependent transformation into material systems. Such structures may change shape, stiffness, or performance in response to environmental conditions or occupancy patterns [67,99]. This approach could redefine adaptability in future buildings and infrastructure [48].

**Figure 14:**
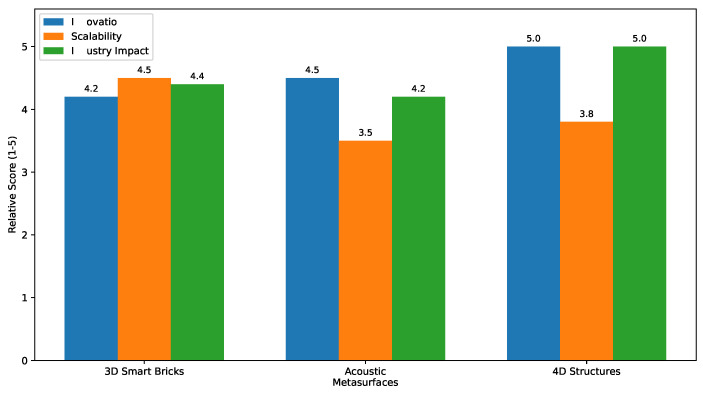
Qualitative comparison of major emerging trends in smart materials for construction, considering innovation level, scalability potential, and expected industry impact. Relative scores were assigned from trends consistently reported in the reviewed literature.

To complement the results summarized in Table 13, Figure 14 compares the relative disruptive potential of the main emerging trends in smart construction materials.

Figure 14 indicates that 4D structures show the highest disruptive potential due to their adaptive behavior, while smart bricks exhibit strong scalability and near-term applicability [48,67,99]. Acoustic metasurfaces also present significant value for urban comfort and environmental quality [69]. These findings suggest that the future of construction materials will increasingly depend on programmable and multifunctional systems [48,51].

## 4. Discussion

The results presented in the previous Section 3 provide a comprehensive overview of the current state of smart materials in the construction industry, highlighting their principal families, functional capabilities, engineering applications, and sustainability contributions. Building on these findings, the following discussion critically examines the implications of the identified trends, explores the principal barriers to large-scale adoption, and outlines future research directions required to accelerate the integration of smart materials into next-generation construction systems.

### 4.1. Synthesis by Material Type and Technological Maturity

The practical implementation of smart materials depends not only on technical performance, but also on their level of technological maturity. Some material families are already entering commercial markets, while others remain at laboratory or pilot scale due to cost, standardization, or production barriers [48,49,64,66]. Evaluating maturity alongside strengths and weaknesses provides a clearer perspective on near-term adoption potential [31,67,68].

Table 14 summarizes the main smart material families according to their current maturity stage, dominant strengths, and principal limitations.

Table 14 shows that smart concretes currently present one of the most direct pathways toward large-scale implementation because they build upon conventional cementitious technologies while adding durability and sensing functions [49,66,68]. SMA have also advanced considerably, especially in seismic retrofitting and high-value infrastructure, although cost and standardization remain limiting factors [31,56,66].

Smart geopolymers demonstrate strong sustainability potential but still require greater consistency in raw materials and broader regulatory acceptance [49,64]. In contrast, PCM-based solutions have already reached niche commercial markets, particularly in energy-efficient buildings, where thermal storage benefits justify their use despite higher design complexity [48,67].

Overall, maturity levels indicate that no single family dominates all dimensions simultaneously. Market penetration is closely linked to balancing performance benefits with manufacturability, cost competitiveness, and code acceptance [31,64,66].

**Figure 15:**
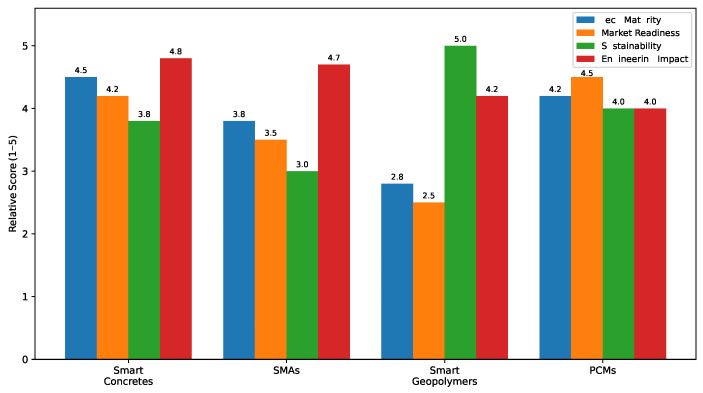
Qualitative comparison of major smart material families in construction according to technological maturity, market readiness, sustainability value, and engineering impact. Relative scores were assigned from trends consistently reported in the reviewed literature.

To complement the synthesis presented in Table 14, Figure 15 compares the relative technological maturity and market readiness of the principal smart material families used in construction.

Figure 15 indicates that smart concretes currently show the strongest balance between maturity and engineering impact, while PCMs lead commercial readiness in building applications [48,66,67]. SMA provide outstanding technical value but remain constrained by cost, whereas smart geopolymers exhibit strong sustainability potential with lower present market maturity [31,56,64].

### 4.2. Performance–Sustainability Balance

The transition toward intelligent construction systems requires balancing engineering performance with environmental and economic sustainability. In practice, two dominant pathways are frequently identified: increasing the incorporation of advanced smart materials, or enhancing digitalization through sensing, automation, and data-driven control [48,66,67,74]. Both strategies can improve infrastructure efficiency, but each involves specific trade-offs [31,49,64].

Table 15 summarizes the comparative benefits and limitations of these two strategic approaches.

Table 15 shows that increasing the use of smart materials can substantially improve structural resilience, durability, and autonomous functionality. These benefits often translate into lower maintenance frequency and fewer unexpected failures over the asset lifecycle [49,66,68]. However, some advanced materials may require greater energy consumption, specialized processing, or scarce resources during manufacturing [31,64].

In contrast, greater digitalization focuses on optimizing existing systems through sensors, predictive maintenance, and real-time control. This pathway can reduce operational waste, improve resource efficiency, and lower long-term costs without necessarily increasing material intensity [48,67,74]. Nevertheless, it introduces dependence on reliable data streams, communication infrastructure, and cybersecurity protection [31,74].

These findings suggest that neither pathway alone is sufficient for future sustainable construction. The most effective strategy is likely a balanced integration of high-value smart materials with robust digital intelligence [48,49,66].

**Figure 16:**
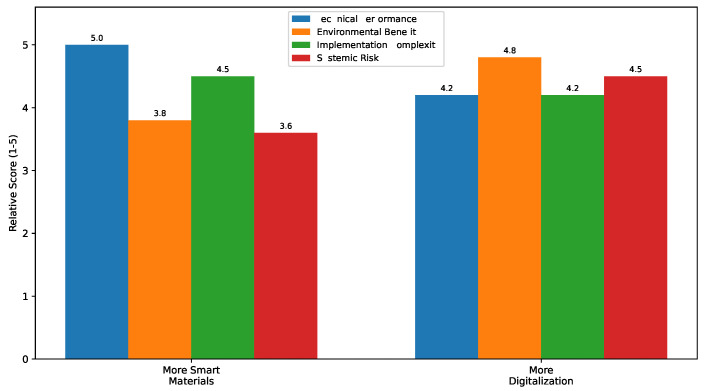
Qualitative comparison between material-intensive and digitalization-intensive pathways for smart construction systems according to technical performance, environmental benefit, implementation complexity, and systemic risk. Relative scores were assigned from trends consistently reported in the reviewed literature.

To complement the synthesis presented in Table 15, Figure 16 compares the relative positioning of both approaches according to technical performance, environmental benefit, implementation complexity, and systemic risk.

Figure 16 indicates that smart-material-intensive strategies offer stronger direct technical gains, whereas digitalization-driven approaches provide superior environmental and operational efficiency [48,66,74]. However, both pathways involve complexity and risk, supporting the need for integrated hybrid solutions [31,67].

### 4.3. Priority Research Gaps

Although smart materials have demonstrated strong potential in construction, several critical knowledge gaps still limit their large-scale and long-term implementation. Existing research is often concentrated on laboratory validation, isolated case studies, or short-term performance indicators, while broader comparative and lifecycle evidence remains limited [31,49,64,66]. Identifying priority research gaps is essential to guide future scientific and industrial development [68,72,74].

Table 16 summarizes the principal unresolved research areas currently highlighted in the literature.

Table 16 shows that long-term durability remains one of the most relevant unresolved issues. Many smart materials have been tested under short laboratory cycles, but evidence covering decades of service exposure is still limited [64,66,68]. Accelerated aging protocols and multiscale predictive models are therefore needed to improve confidence in lifecycle performance [31,66].

Another major gap concerns comparative life cycle assessment (LCA). While many studies report environmental benefits for specific technologies, cross-comparisons among smart concretes, SMAs, PCMs, geopolymers, and digitalized systems remain scarce [49,64,72]. More standardized and transparent LCA frameworks are necessary for robust decision-making [31,72].

The integration of artificial intelligence with smart materials is also at an early stage. Current research includes promising concepts, but few validated systems combine material selection, monitoring, maintenance planning, and sustainability objectives within a unified framework [49,74]. This area offers strong potential for future interdisciplinary progress [66,74].

**Figure 17:**
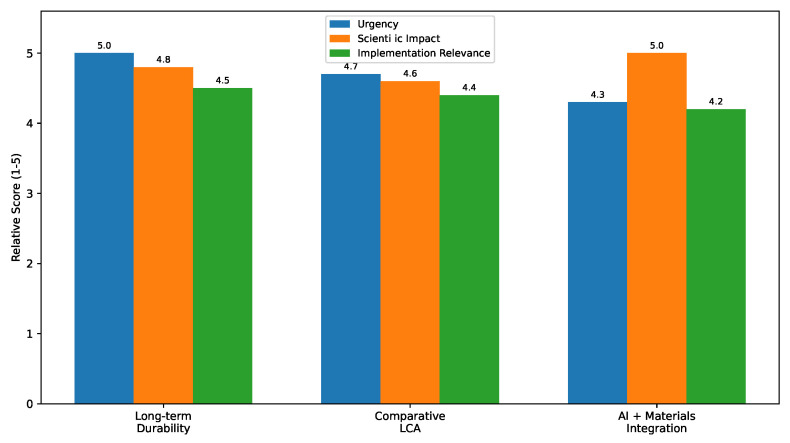
Qualitative comparison of priority research gaps in smart materials for construction according to urgency, expected scientific impact, and implementation relevance. Relative scores were assigned from trends consistently reported in the reviewed literature.

To complement the synthesis presented in Table 16, Figure 17 compares the relative urgency and expected impact of the principal research gaps identified for smart materials in construction.

Figure 17 indicates that long-term durability and comparative lifecycle assessment represent the most urgent priorities, while AI-material integration offers particularly high transformative potential [66,72,74]. These findings suggest that future research should combine materials science, digital engineering, and sustainability assessment in a coordinated manner [31,49].

### 4.4. Perspectives for Large-Scale Implementation

The transition of smart materials from pilot projects to widespread market adoption depends on more than technological readiness. Large-scale implementation requires coordinated regulatory support, economic incentives, and visible evidence of real-world performance [31,49,72,73]. Without these enabling conditions, even technically mature solutions may remain confined to niche applications [56,66,68].

Table 17 summarizes the principal implementation levers that could accelerate the adoption of smart materials in construction.

Table 17 shows that standards and technical codes are among the most influential catalysts for adoption. Clear design procedures and certification pathways can reduce uncertainty for engineers, insurers, and public agencies, especially for emerging technologies such as smart alloys, monitoring systems, and geopolymer materials [31,49,73].

Economic instruments are also important. Tax incentives, subsidies, and carbon-related financing can offset the higher upfront costs often associated with innovative materials [49,72]. This is particularly relevant when lifecycle benefits are strong but not immediately reflected in procurement decisions [31].

Full-scale demonstrator projects may provide the most persuasive evidence for market transformation. Pilot bridges, buildings, and infrastructure systems equipped with long-term monitoring can validate durability, cost-effectiveness, and user acceptance under real operating conditions [56,66,68].

These findings suggest that successful deployment of smart materials will depend on combining policy support, economic mechanisms, and visible engineering proof [31,66,72].

**Figure 18:**
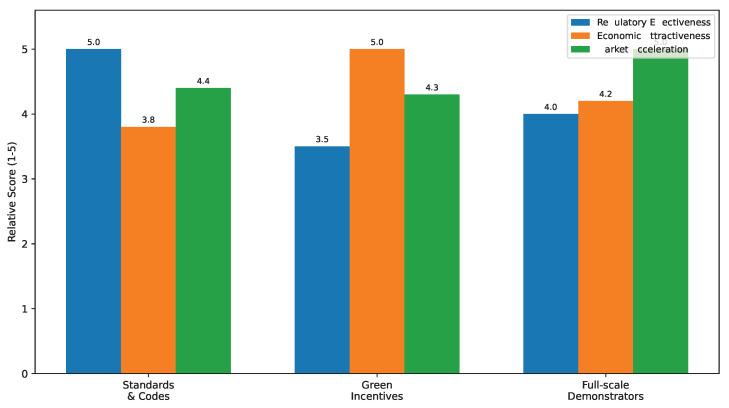
Qualitative comparison of principal implementation levers for large-scale adoption of smart materials in construction, considering regulatory effectiveness, economic attractiveness, and market acceleration potential. Relative scores were assigned from trends consistently reported in the reviewed literature.

To complement the synthesis presented in Table 17, Figure 18 compares the relative influence of the main levers for accelerating large-scale adoption of smart materials in construction.

Figure 18 indicates that full-scale demonstrators offer the strongest market acceleration effect, while standards and codes provide the highest regulatory confidence [31,66,68]. Green incentives also play a critical enabling role by improving short-term financial feasibility [49,72]. Together, these mechanisms can significantly shorten the path toward mainstream adoption [56,73].

### 4.5. Positioning of This Review Within the State-of-the-Art Literature

To contextualize the contribution of the present study within the rapidly expanding body of knowledge on smart materials in construction, a comparative assessment of representative review papers was conducted. Previous studies have examined specific domains such as self-healing materials, SMA, piezoelectric sensing systems, sustainable smart materials, energy-efficient envelopes, and digital construction technologies. However, most available reviews remain technologically fragmented, focusing on individual material families or isolated performance dimensions.

A comprehensive integration of structural functionality, sustainability performance, digital connectivity, lifecycle benefits, and implementation barriers is still limited in the literature. The present review addresses this gap through a PRISMA-based systematic framework that simultaneously evaluates smart material categories, quantitative engineering performance, architectural and structural applications, environmental benefits, IoT integration, and market readiness.

Table 18 compares representative state-of-the-art reviews and highlights the positioning of this study.

While previous reviews provide valuable insights into specific smart material families or application niches, few studies simultaneously evaluate mechanical performance, thermal efficiency, sensing capability, lifecycle sustainability, and deployment feasibility. The present review advances the field by linking material intelligence with practical construction outcomes and transition pathways toward next-generation infrastructure.

To further clarify thematic coverage, Table 19 compares the extent to which representative reviews address key smart material domains and evaluation dimensions. For transparency, the evaluated categories were operationally defined as follows: Concrete refers to smart concretes, cementitious systems, and geopolymer-based materials; SMA denotes shape memory alloys and adaptive deformation systems; Sensors includes self-sensing, piezoelectric, conductive, and structural monitoring technologies; Thermal refers to PCM systems, adaptive thermal materials, and building-energy regulation; ESG covers sustainability, environmental performance, and decarbonization considerations; IoT/AI includes digital integration, intelligent monitoring, artificial intelligence, and smart-construction ecosystems; Barriers denotes discussion of implementation, scalability, regulatory, or market limitations; and Lifecycle refers to lifecycle assessment, durability, lifecycle costing, or lifecycle performance considerations.

The thematic comparison confirms that existing reviews often specialize in either material science mechanisms, sustainability aspects, or digital innovation. In contrast, this study simultaneously addresses smart concretes, SMA, sensing technologies, adaptive thermal systems, environmental performance, lifecycle impacts, economic barriers, and intelligent digital integration.

Importantly, the present review is aligned with the Scopus search window applied in the methodology (2021–2026), thereby emphasizing the most recent stage of development in smart construction materials. This period captures the accelerated emergence of AI-assisted design, digital twins, self-sensing UHPC, multifunctional geopolymers, advanced PCM systems, and carbon-oriented material innovation.

This broader positioning enables a systems-oriented interpretation of smart materials in construction, emphasizing that successful adoption depends not only on intrinsic material properties, but also on manufacturability, code acceptance, lifecycle economics, interoperability with digital platforms, and contextual sustainability objectives.

By bridging these domains, the present review moves beyond technology-centric descriptions and supports the development of resilient, data-driven, and low-carbon built environments based on multifunctional smart material systems.

## 5. Conclusions

Smart materials are progressively transforming the construction sector by shifting conventional passive materials toward multifunctional systems capable of sensing, adaptation, self-repair, energy regulation, and enhanced lifecycle performance. The evidence synthesized through this PRISMA-based systematic review confirms that these technologies are no longer limited to experimental concepts, but are increasingly emerging as strategic components for resilient, sustainable, and digitally connected buildings and infrastructure.

The reviewed literature demonstrates that different smart material families contribute through complementary engineering functions. Self-healing concretes and intelligent cementitious systems improve durability and reduce maintenance interventions; piezoelectric and conductive materials enable structural health monitoring and decentralized sensing; SMA and rheological systems enhance seismic resilience and vibration control; while phase change materials, thermochromic systems, and adaptive envelopes improve thermal efficiency and indoor comfort. This multifunctional diversity indicates that future high-performance construction systems will likely depend on hybrid combinations of several smart material technologies rather than isolated solutions.

From a sustainability perspective, smart materials show significant potential to support decarbonization and resource efficiency across multiple lifecycle stages. Geopolymers, recycled materials, additive manufacturing, PCM-integrated envelopes, and durable self-healing systems can reduce embodied emissions, operational energy demand, maintenance frequency, and construction waste. Therefore, the contribution of smart materials extends beyond technical innovation toward broader environmental and socioeconomic objectives aligned with next-generation low-carbon construction.

The review also confirms that the full value of smart materials is amplified when integrated with digital technologies such as IoT platforms, artificial intelligence, wireless sensing networks, BIM environments, and digital twins. This convergence enables real-time monitoring, predictive maintenance, data-driven optimization, and smarter asset management. Consequently, the future of construction intelligence will likely arise from the interaction between material intelligence and digital intelligence rather than from either domain independently.

Despite these advances, several barriers continue to restrict large-scale implementation. High initial costs, manufacturing scalability, durability uncertainty under long-term service conditions, lack of technical standards, regulatory delays, and limited market confidence remain major constraints. Overcoming these challenges will require coordinated progress in industrial production, code development, economic incentives, cybersecurity readiness, and full-scale demonstrator projects capable of validating performance under real operating conditions.

The comparative analysis further suggests that technological maturity is uneven across material families. Smart concretes, PCM-based systems, and selected sensing technologies show stronger short-term implementation readiness, whereas smart geopolymers, advanced nanocomposites, autonomous healing systems, and highly adaptive 4D structures remain at earlier stages of commercialization despite strong long-term potential. This finding highlights the importance of aligning research priorities with both technical promise and market feasibility.

Several priority research needs were identified. Future studies should strengthen long-term durability assessment, standardized lifecycle comparison among competing technologies, interoperability with digital ecosystems, cost-benefit quantification, and multi-objective optimization frameworks integrating structural, environmental, and economic criteria. Greater availability of field data from bridges, buildings, pavements, and retrofit projects will be essential to accelerate evidence-based adoption.

The preparation of this review was motivated by the increasing diversification of smart material technologies in construction and by the limited availability of integrative comparative analyses connecting engineering functionality, sustainability performance, and implementation maturity across material families. By synthesizing these complementary dimensions within a unified analytical perspective, this work seeks to support future research prioritization, technology assessment, and evidence-based decision-making for next-generation built environments.

Overall, smart materials represent one of the most promising pathways toward safer, longer-lasting, lower-carbon, and more adaptive built environments. Their successful deployment, however, will depend not only on intrinsic material performance, but also on manufacturability, affordability, regulatory acceptance, and intelligent system integration. As construction moves toward sustainability, resilience, and automation, smart materials are expected to become foundational elements of next-generation infrastructure systems.

## Figures and Tables

**Table 1:** Taxonomic classification of major smart material families used in construction according to operating principles, representative examples, and engineering applications.

Smart Material Type	Operating Principle	Representative Examples	Applications	References
Self-healing materials	Chemical or biological reaction after damage	Healing polymers, bacterial binders	Crack repair, service-life extension	[49,51,54]
Smart concretes	Nanotechnology or conductive response under loading	Self-sensing concrete, nano-modified concrete	Structural monitoring, durability enhancement	[30,47,48,53]
Piezoelectric materials	Electric charge generation under deformation	PZT, PVDF, embedded sensors	Structural monitoring, vibration sensing, energy harvesting	[46,47,48,55,56]
Thermochromic materials	Optical change with temperature variation	Smart glazing, thermochromic coatings	Adaptive façades, solar control	[50,57,58]
Adaptive thermal materials	Heat storage or phase transition response	PCMs, thermal panels	Thermal efficiency, indoor comfort	[48,50,59]
Magnetorheological materials	Viscosity change under magnetic field	MR fluids	Seismic dampers, vibration control	[46,55,56]
Electrorheological materials	Viscosity change under electric field	ER fluids	Adaptive devices, controllable dampers	[46,55]
Shape memory materials	Recovery of original shape after thermal or stress stimulus	NiTi alloys, Fe-SMA, SMP polymers	Seismic retrofitting, prestressing, actuators	[31,46,56,60,61]
Conductive nanocomposites	Conductivity variation under external stimulus	CNT-doped concrete, graphene composites	Self-diagnosis, crack sensing, radiant heating	[30,47,52,62,63]
Multifunctional geopolymers	Functionalized low-carbon binders from industrial residues	Waste-based geopolymer composites	Self-sensing, self-cleaning, sustainable construction	[64,65]

**Table 2:** Functional implementation framework of major smart material families used in construction, including representative construction examples, dominant stimuli, and typical engineering responses.

Family/Category	Construction Examples	Main Stimulus	Typical Functional Response	References
Smart concretes	Self-healing concrete, self-sensing concrete, self-monitoring UHPC	Cracks, load, humidity	Self-repair, resistivity change, SHM	[49,61,64,66,68,69,70]
SMA	NiTi, Fe-SMA, Cu-SMA	Temperature, deformation	Shape recovery, seismic dissipation	[31,46,56,61,66]
Piezoelectric materials	PZT, triangular pavement transducers	Deformation, vibration	Voltage generation, sensing, energy harvesting	[31,46,66,67,68,71]
Phase change materials (PCM)	PCM walls, bricks, façades	Temperature	Heat storage/release, thermal regulation	[48,49,67,69]
Smart geopolymers	Self-sensing, self-compacting, self-cleaning geopolymers	Load, fouling	Electrical change, self-cleaning, self-heating	[49,64]
Smart rheological fluids	MR and ER damping devices	Magnetic/electric field	Rapid viscosity and stiffness change	[46,55,56,66]
Smart coatings/smart bricks	PCM bricks, graphene/PLA bricks, photocatalytic coatings	Radiation, water, dirt	Self-cleaning, thermal enhancement, conductive sensing	[48,49,51,69]

**Table 3:** Functional properties and reported quantitative performance ranges of smart materials used in construction.

Functional Property	Reported Range / Value	Representative Materials	References
Microstrain detection sensitivity	Up to 10 µϵ in self-sensing concrete	Self-sensing concrete, sensing UHPC	[48,68,71]
Fracture toughness improvement	≈50% over reference concrete	Fiber-reinforced concrete, nano-additives	[48,64,73]
Corrosion resistance	≈40% higher than conventional steel	SMAs, smart protective coatings	[31,48]
Fire stability	Up to 1200 °C in adaptive composites	Smart bricks, advanced composites	[48]
Conductivity for sensing	≈10 S/m with embedded electrodes	Smart bricks, graphene/CNT composites	[48,64,69]
Wall heat flux reduction	Average decrease of ≈45%	PCM-integrated walls	[31,67]
Carbon footprint reduction	40–60% CO_2_ reduction using AM + green materials	3D printing, geopolymers, smart bricks	[48,49,64,72,74]

**Table 4:** Main structural and architectural applications of smart materials in construction.

Application Domain	Smart Solution	Main Function	Benefit	References
SHM in bridges/buildings	Self-sensing concrete, optical fiber, PZT sensors	Real-time monitoring	Early damage detection, improved safety	[31,46,61,66,68,71]
Seismic protection	SMAs, MR/ER dissipative devices	Structural response control	Damage reduction, resilience	[31,56,66]
Energy-harvesting pavements	Piezoelectric devices under roadways	Traffic energy harvesting	Local renewable energy generation	[56,67,68]
Climate-responsive façades/walls	PCMs, chromogenic systems, smart bricks	Passive thermal/light management	Energy savings, indoor comfort	[48,49,67,69]
Autonomous crack healing	Bacterial/self-healing concrete	Self-sealing of fissures	Longer service life, lower maintenance	[49,61,66,70]
Strengthening/rehabilitation	SMAs, smart FRP composites	Post-strengthening of existing structures	Reduced intervention, seismic sustainability	[31,56]

**Table 5:** Main categories of smart concretes and self-sensing UHPC systems used in construction.

Smart Concrete Type	Main Mechanism	Advantages	Limitations	References
Self-healing concrete (bacterial/polymeric)	Limestone precipitation or healing-agent release	Crack reduction, lower repair demand	Cost, activation control	[49,61,66,70]
Conventional self-sensing concrete	Carbon fibers, CNTs, carbon black nanofillers	Distributed SHM, reduced instrumentation	Homogeneity, additive cost	[68,71,73]
Self-sensing UHPC	Dense matrix with conductive reinforcement	High strength and damage sensitivity	Large-scale production, cost	[68]
Smart geopolymer concrete	Alkali-activated matrix with conductive residues	Low CO_2_ footprint, multifunctionality	Residue variability, standards	[49,64]

**Table 6:** Main sustainability strategies and environmental performance of smart materials in construction.

Strategy	Technology/Material	Environmental/Economic Effect	References
OPC substitution	Geopolymers, SCMs, green binders	Major reduction of cement-related CO_2_ emissions	[49,64,72]
Additive manufacturing (AM/3D)	3D-printed concrete, smart bricks	∼50% less waste, 40–60% lower carbon footprint	[48,49,72]
Energy efficiency	PCMs, adaptive envelopes	Up to 30–40% lower energy consumption	[48,49,67,69]
Green cities	Smart concrete, SMA, metasurfaces	Lower maintenance, improved acoustic comfort	[31,69]
AI optimization	AI frameworks + smart materials	30% lower cost, 25% less waste, 40% higher efficiency	[74]

**Table 7:** Main smart material families contributing to carbon reduction in construction across lifecycle stages.

Type/Family	Key Examples	Main CO_2_ Reduction Mechanism	Lifecycle Stage Impacted	References
Advanced concretes	Self-healing, photocatalytic, low-clinker concrete	Lower cement demand, self-cleaning, CO_2_ capture	Production, use, end-of-life	[48,49,79,80,81,82,83]
Bio-based materials	CLT timber, bamboo, biopolymers	Biogenic carbon storage, low embodied energy	Production, use	[75,77,82,84,85,86,87]
Nanomaterials	Nanoclays, nanosilica, nanocomposites	Improved performance with less material use	Production, use	[48,88,89,90]
Recycled materials	Recycled steel, recycled aggregates	Lower manufacturing energy and emissions	Production	[76,78,91,92]
Phase change materials (PCMs)	PCM walls, PCM bricks	Lower operational energy demand	Use	[48,89,90,93,94,95]
Smart envelopes	Aerogels, electrochromic glazing	Superior insulation and solar control	Use	[90,93,94,95,96]
Smart prefabricated systems	SMART frame structures, modular systems	Material optimization and lower construction waste	Construction	[97,98]

**Table 8:** Technical properties of smart materials associated with carbon reduction and circular performance.

Property/Function	Typical Improvement	Leading Materials	Carbon Impact	References
Low embodied energy	Up to 40% lower than conventional materials	Biomaterials, geopolymers	Lower initial footprint	[75,77,80,82,84]
Biogenic sequestration	Up to 60% lower net emissions	CLT and laminated timber	Temporary CO_2_ storage	[75,77,85,86]
Self-healing capacity	Up to 50% longer service life	Self-healing concretes	Fewer replacements and repairs	[49,79]
Ultra-low thermal conductivity	Up to 80% lower than traditional concrete	Aerogels, nanoclays, PCMs	Reduced operational energy demand	[88,93,95]
Recyclability	Above 90% potential recovery	Recycled steel, prefabricated systems	Circularity and lower waste	[78,92]

**Table 9:** Main digital support technologies integrated with smart materials in construction.

Support Technology	Role with Smart Materials	Benefit	References
IoT + SHM	Connected embedded sensors (Zigbee, LoRa, 5G)	Real-time data for maintenance decisions	[46,48,67,74]
AI/ML	Optimal material selection and predictive maintenance	Failure reduction, cost and energy optimization	[49,74]
Digital twin/BIM	Behavioral models of smart materials and systems	Lifecycle design and asset management	[66,74,99]

**Table 10:** Main technical and economic challenges limiting the adoption of smart materials in construction.

Challenge Type	Manifestation in Smart Materials	Impact on Adoption	References
High initial cost	SMAs, nanomaterials, advanced sensing systems	Limits large-scale implementation	[31,64,66,68,73,74]
Scalability	Production of self-sensing UHPC, multifunctional geopolymers	Difficult industrialization	[49,64,66,68]
Compatibility	Integration with conventional systems and in situ processes	Risk of failures, cost overruns	[31,48,67,73]
Durability/stability	Nanomaterial degradation, NiTi corrosion, aging effects	Long-term uncertainty	[31,64,68]

**Table 11:** Main regulatory, security, and market acceptance challenges affecting smart materials in construction.

Dimension	Identified Problem	Main Consequence	References
Regulation	Lack of codes for SMAs, geopolymers, and SHM systems	Difficult design approval and certification	[31,49,64,73]
Safety/Cybersecurity	Risk of cyberattacks on embedded systems	Threat to operational reliability	[48,74]
Market acceptance	Perceived risk and uncertainty about service life	Resistance from developers and users	[31,72,73]

**Table 12:** Main impacts of smart materials on structural sustainability, resilience, and lifecycle performance.

Sustainability Aspect	Material/Solution	Effect on Structural Sustainability	References
Seismic resilience	SMAs, MR/ER devices, smart dampers	Less damage, fewer repairs, higher social safety	[31,56]
Lifecycle costs	Self-healing concrete, SHM systems	Strong reduction of maintenance and unexpected failures	[31,49,61,66,68,70]
Sustainable rehabilitation	SMA strengthening systems, smart composites	Lower intervention intensity, reduced footprint in historic structures	[31,56]

**Table 13:** Main emerging trends in smart materials for construction and their potential impact.

Trend	Brief Description	Potential Impact	References
3D smart bricks	Bricks incorporating PCM, graphene, or embedded electronics	Highly efficient modular buildings	[48,51]
Acoustic metasurfaces	Programmable surfaces for sound control	Quieter and more comfortable cities	[69]
4D structures	Integration of time as a functional design dimension	Active adaptation to climate and use conditions	[48,67,99]

**Table 14:** Synthesis of smart material families according to technological maturity, strengths, and key limitations.

Family	Maturity (Lab ↔ Market)	Dominant Strengths	Main Weaknesses	References
Smart concretes	Demonstration → implementation	Strong direct impact on durability	Cost, homogeneous additive control	[49,61,66,68,70]
SMAs	Pilot projects → commercial niches	Exceptional seismic performance	NiTi cost, corrosion, lack of codes	[31,56,66]
Smart geopolymers	Laboratory → pilot scale	Very low CO_2_ footprint, multifunctionality	Residue variability, standardization	[49,64]
PCMs in buildings	Commercial in niche markets	Significant energy savings	Complex thermal design, cost	[48,49,67]

**Table 15:** Comparison between material-intensive and digitalization-intensive pathways for smart construction systems.

Approach	Main Technical Benefit	Key Environmental/Economic Benefit	Relevant Trade-Offs	References
More smart materials	Higher resilience, multifunctionality, structural performance	Fewer failures and repair interventions	Higher embodied energy and material resource demand	[31,49,64,66,68]
More digitalization	Real-time optimization and predictive control	Lower waste generation and operating costs	Cybersecurity risks, dependence on data quality	[31,48,67,74]

**Table 16:** Priority research gaps for future development of smart materials in construction.

Research Area	Current Gap	Future Need	References
Long-term durability	Scarcity of studies beyond 20–30 simulated years	Multiscale models and accelerated testing methods	[31,64,66,68]
Comparative LCA across technologies	Many isolated case studies	Systematic comparisons among material families	[31,49,64,72]
AI + materials integration	Few validated integrated proposals	Robust multi-objective decision frameworks	[49,66,74]

**Table 17:** Main implementation levers for large-scale deployment of smart materials in construction.

Implementation Lever	Suggested Actions	Expected Impact	References
Standards and codes	Specific guidelines for SMAs, SHM systems, geopolymers	Reduction of perceived risk and easier certification	[31,49,73]
Green incentives	Tax credits, subsidies, carbon finance instruments	Compensation of higher initial costs	[31,49,72]
Full-scale demonstrators	Bridges and buildings with monitored pilot deployment	Evidence of performance and social acceptance	[31,56,66,68]

**Table 18:** Comparison of representative reviews on smart materials in construction and the positioning of this study.

Work	Main Focus	Methodology	Strengths	Limitations	Contribution of This Work
[51]	Smart materials in civil engineering applications	Narrative review	Broad early overview of multifunctional materials	Limited sustainability and quantitative comparison	Extends toward lifecycle performance, decarbonization, and measurable engineering indicators
[31]	Sustainability potential of smart materials in buildings	Critical review	Strong environmental and resilience perspective	Limited coverage of sensing systems and digital integration	Integrates sustainability with SHM, IoT, and multifunctional infrastructure applications
[46]	Piezoelectric, rheological, and adaptive materials	Technical review	Detailed explanation of responsive mechanisms	Limited architectural and market implementation analysis	Connects functional mechanisms with real construction deployment pathways
[49]	Smart and sustainable cementitious materials	Review of advanced concrete systems	Strong focus on self-healing and low-carbon binders	Primarily centered on concrete technologies	Expands scope to façades, SMAs, PCM systems, coatings, and digital ecosystems
[48]	Adaptive materials and future intelligent buildings	Emerging technology review	Forward-looking perspective on responsive envelopes and AI	Limited systematic comparison across mature technologies	Provides evidence-based synthesis across mature and emerging smart materials
This Work	Material families, engineering properties, applications, sustainability, carbon reduction, IoT integration, barriers, and future trends	PRISMA-based systematic review with integrative synthesis	Multi-dimensional comparison across structural, environmental, digital, and economic criteria	Focus on peer-reviewed journal literature	Provides unified framework for intelligent, resilient, and low-carbon construction systems

**Table 19:** Thematic coverage comparison between this review and representative smart materials in construction reviews. Symbol “X” indicates explicit substantive coverage of the corresponding thematic category within the reviewed work.

Work	Concrete	SMA	Sensors	Thermal	ESG	IoT/AI	Barriers	Lifecycle	Period
[51]	X	X	X						Pre-2021
[31]	X	X		X	X		X	X	2021
[46]		X	X						2021
[49]	X		X		X		X	X	2023
[48]	X	X	X	X	X	X		X	2025
This Work	X	X	X	X	X	X	X	X	2021–2026

## Data Availability

All data generated or analyzed during this study are included in this published article.

## Supplementary Materials

The following supporting information can be downloaded at: https://www.mdpi.com/article/10.3390/ma19122676/s1. The Supplementary Materials include the PRISMA 2020 checklist. Reference [33] is cited in the Supplementary Materials.

## Abbreviations

The following abbreviations are used in this manuscript:

AI	Artificial Intelligence
AM	Additive Manufacturing
BIM	Building Information Modeling
CF	Carbon Footprint Factor (carbon intensity)
CLT	Cross-Laminated Timber
CNT	Carbon Nanotube
CO_2_	Carbon Dioxide
Cu-SMA	Copper-Based Shape Memory Alloy
ER	Electrorheological
ESG	Environmental, Social, and Governance
FCR	Fractional Change in Resistance
Fe-SMA	Iron-Based Shape Memory Alloy
FRP	Fiber-Reinforced Polymer
HE	Healing Efficiency
HVAC	Heating, Ventilation, and Air Conditioning
IoT	Internet of Things
LCA	Life Cycle Assessment
LCCE	Lifecycle Cost-Effectiveness
ML	Machine Learning
MR	Magnetorheological
NiTi	Nickel–Titanium
OPC	Ordinary Portland Cement
OSF	Open Science Framework
PCM	Phase Change Material
PLA	Polylactic Acid
PRISMA	Preferred Reporting Items for Systematic Reviews and Meta-Analyses
PVDF	Polyvinylidene Fluoride
PZT	Lead Zirconate Titanate
RoB 2	Risk of Bias 2 (tool)
ROBINS-I	Risk of Bias in Non-Randomized Studies of Interventions
SCM	Supplementary Cementitious Material
SHM	Structural Health Monitoring
SMA	Shape Memory Alloy
SMP	Shape Memory Polymer
TRL	Technology Readiness Level
UHPC	Ultra-High-Performance Concrete
