# Supplementary material for: Smart Materials Employed in the Construction Industry: A Systematic Review of Types, Properties, Applications, and Sustainability Performance
Source: Materials (Basel). 2026 Jun 22;19(12):2676. doi: 10.3390/ma19122676 (PMC13304312; doi:10.3390/ma19122676)
Supplement: Supplementary file 1 [file materials-19-02676-s001.zip › materials-4362461-supplementary.pdf]

PRISMA 2020 Checklist

Section and Topic	Item #	Checklist item	Location where item is reported
**TITLE**			
Title	1	Identify the report as a systematic review.	Title page and Abstract (Page 1): "A Systematic Review".
**ABSTRACT**			
Abstract	2	See the PRISMA 2020 for Abstracts checklist.	Abstract (Page 1).
**INTRODUCTION**			
Rationale	3	Describe the rationale for the review in the context of existing knowledge.	Introduction (Pages 1–3, lines 26–92).
Objectives	4	Provide an explicit statement of the objective(s) or question(s) the review addresses.	Introduction (Pages 2–3, lines 81–102).
**METHODS**			
Eligibility criteria	5	Specify the inclusion and exclusion criteria for the review and how studies were grouped for the syntheses.	Sections 2.1–2.4 (Pages 3–6), especially Section 2.3 Study Selection Process and Section 2.4 Study Quality and Robustness Assessment.
Information sources	6	Specify all databases, registers, websites, organisations, reference lists and other sources searched or consulted to identify studies. Specify the date when each source was last searched or consulted.	Section 2.2 Literature Search Strategy (Pages 4–5). Scopus database and OSF protocol repository described.
Search strategy	7	Present the full search strategies for all databases, registers and websites, including any filters and limits used.	Section 2.2 Literature Search Strategy (Pages 4–5, lines 154–162).
Selection process	8	Specify the methods used to decide whether a study met the inclusion criteria of the review, including how many reviewers screened each record and each report retrieved, whether they worked independently, and if applicable, details of automation tools used in the process.	Section 2.3 Study Selection Process Based on PRISMA (Pages 4–5). Sequential screening of titles, abstracts, and full texts described.
Data collection process	9	Specify the methods used to collect data from reports, including how many reviewers collected data from each report, whether they worked independently, any processes for obtaining or confirming data from study investigators, and if applicable, details of automation tools used in the process.	Section 2 Materials and Methods (Pages 3–9). Comparative extraction of engineering indicators and bibliometric information described; no automation tools reported.
Data items	10a	List and define all outcomes for which data were sought. Specify whether all results that were compatible with each outcome domain in each study were sought (e.g. for all measures, time points, analyses), and if not, the methods used to decide which results to collect.	Sections 2.5 and 2.6 (Pages 6–9). Outcomes included structural performance, sensing capability, thermal efficiency, sustainability indicators, durability, lifecycle performance, and technological maturity.
	10b	List and define all other variables for which data were sought (e.g. participant and intervention characteristics, funding sources). Describe any assumptions made about any missing or unclear information.	Sections 2.4–2.6 (Pages 5–9). Variables included material family, application domain, engineering indicators, sustainability contribution, and implementation barriers. Studies lacking methodological transparency were excluded.

PRISMA 2020 Checklist

Section and Topic	Item #	Checklist item	Location where item is reported
Study risk of bias assessment	11	Specify the methods used to assess risk of bias in the included studies, including details of the tool(s) used, how many reviewers assessed each study and whether they worked independently, and if applicable, details of automation tools used in the process.	Section 2.4 Study Quality and Robustness Assessment (Pages 5–6). Qualitative robustness assessment used; no formal quantitative risk-of-bias tool applied due to heterogeneity.
Effect measures	12	Specify for each outcome the effect measure(s) (e.g. risk ratio, mean difference) used in the synthesis or presentation of results.	Section 2.6 Analytical and Conceptual Framework (Pages 8–9), Equations (1)–(7).
Synthesis methods	13a	Describe the processes used to decide which studies were eligible for each synthesis (e.g. tabulating the study intervention characteristics and comparing against the planned groups for each synthesis (item #5)).	Sections 2.3 and 2.4 (Pages 4–6). Studies selected based on thematic relevance, methodological transparency, and availability of comparable engineering data.
	13b	Describe any methods required to prepare the data for presentation or synthesis, such as handling of missing summary statistics, or data conversions.	Sections 2.5 and 2.6 (Pages 6–9). Structured qualitative and comparative synthesis adopted due to heterogeneity.
	13c	Describe any methods used to tabulate or visually display results of individual studies and syntheses.	Results section tables and figures (Figures 1–7; Tables 1–6). Bibliometric mapping performed with VOSviewer.
	13d	Describe any methods used to synthesize results and provide a rationale for the choice(s). If meta-analysis was performed, describe the model(s), method(s) to identify the presence and extent of statistical heterogeneity, and software package(s) used.	Sections 2.5 and 2.6 (Pages 6–9). No meta-analysis conducted because of heterogeneity; qualitative comparative synthesis performed.
	13e	Describe any methods used to explore possible causes of heterogeneity among study results (e.g. subgroup analysis, meta-regression).	Sections 2.5 and 2.6 (Pages 7–9). Heterogeneity discussed in terms of specimen scale, material composition, testing methods, and environmental exposure.
	13f	Describe any sensitivity analyses conducted to assess robustness of the synthesized results.	Not applicable. No quantitative meta-analysis or sensitivity analysis was conducted.
Reporting bias assessment	14	Describe any methods used to assess risk of bias due to missing results in a synthesis (arising from reporting biases).	Not formally assessed; acknowledged as a limitation in Sections 2.4 and Discussion.
Certainty assessment	15	Describe any methods used to assess certainty (or confidence) in the body of evidence for an outcome.	Qualitative robustness assessment described in Section 2.4 (Pages 5–6).
**RESULTS**			
Study selection	16a	Describe the results of the search and selection process, from the number of records identified in the search to the number of studies included in the review, ideally using a flow diagram.	Section 2.3 and Figure 1 (Pages 4–5).
	16b	Cite studies that might appear to meet the inclusion criteria, but which were excluded, and explain why they were excluded.	Section 2.3 (Pages 4–5). Exclusion reasons summarized during screening

PRISMA

PRISMA 2020 Checklist

Section and Topic	Item #	Checklist item	Location where item is reported
			and eligibility phases.
Study characteristics	17	Cite each included study and present its characteristics.	Results section Tables 1–6 and corresponding discussion (Pages 10–19).
Risk of bias in studies	18	Present assessments of risk of bias for each included study.	Section 2.4 (Pages 5–6). Qualitative assessment approach reported.
Results of individual studies	19	For all outcomes, present, for each study: (a) summary statistics for each group (where appropriate) and (b) an effect estimate and its precision (e.g. confidence/credible interval), ideally using structured tables or plots.	Results section Tables 1–6 and Figures 3–7 (Pages 10–19).
Results of syntheses	20a	For each synthesis, briefly summarise the characteristics and risk of bias among contributing studies.	Sections 3.1–3.6 (Pages 10–19).
	20b	Present results of all statistical syntheses conducted. If meta-analysis was done, present for each the summary estimate and its precision (e.g. confidence/credible interval) and measures of statistical heterogeneity. If comparing groups, describe the direction of the effect.	No statistical meta-analysis conducted due to heterogeneity; qualitative comparative synthesis reported in Sections 3.1–3.6.
	20c	Present results of all investigations of possible causes of heterogeneity among study results.	Sections 2.5 and 2.6 discuss heterogeneity sources (Pages 7–9).
	20d	Present results of all sensitivity analyses conducted to assess the robustness of the synthesized results.	Not applicable.
Reporting biases	21	Present assessments of risk of bias due to missing results (arising from reporting biases) for each synthesis assessed.	Not formally evaluated; acknowledged as limitation.
Certainty of evidence	22	Present assessments of certainty (or confidence) in the body of evidence for each outcome assessed.	Section 2.4 and Discussion sections.
**DISCUSSION**			
Discussion	23a	Provide a general interpretation of the results in the context of other evidence.	Discussion section (Section 4).
	23b	Discuss any limitations of the evidence included in the review.	Discussion section and Sections 2.4–2.6.
	23c	Discuss any limitations of the review processes used.	Sections 2.1, 2.2, and Discussion. Single-database limitation and retrospective protocol registration acknowledged.
	23d	Discuss implications of the results for practice, policy, and future research.	Discussion and Conclusions sections (Sections 4 and 5).
**OTHER INFORMATION**			
Registration and protocol	24a	Provide registration information for the review, including register name and registration number, or state that the review was not registered.	Section 2.1 PRISMA Systematic Review Protocol (Page 3): OSF registration https://doi.org/10.17605/OSF.IO/Z3XJ4
	24b	Indicate where the review protocol can be accessed, or state that a protocol was not prepared.	Section 2.1 (Page 3): OSF repository link provided.
	24c	Describe and explain any amendments to information provided at registration or in the protocol.	Section 2.1 (Page 3). Retrospective registration acknowledged as methodological limitation.

PRISMA

PRISMA 2020 Checklist

Section and Topic	Item #	Checklist item	Location where item is reported
Support	25	Describe sources of financial or non-financial support for the review, and the role of the funders or sponsors in the review.	The authors declare that no external financial support was received for the development of this systematic review. Institutional academic support was provided by the Facultad de Ingeniería, Universidad Autónoma de Querétaro.
Competing interests	26	Declare any competing interests of review authors.	The authors declare no conflict of interest related to this study.
Availability of data, code and other materials	27	Report which of the following are publicly available and where they can be found: template data collection forms; data extracted from included studies; data used for all analyses; analytic code; any other materials used in the review.	OSF repository described in Section 2.1; supplementary material may include checklist and review protocol.

From: Page MJ, McKenzie JE, Bossuyt PM, Boutron I, Hoffmann TC, Mulrow CD, et al. The PRISMA 2020 statement: an updated guideline for reporting systematic reviews. BMJ 2021;372:n71. doi: 10.1136/bmj.n71.
This work is licensed under CC BY 4.0. To view a copy of this license, visit https://creativecommons.org/licenses/by/4.0/
